# The Conserved ADAMTS-like Protein Lonely heart Mediates Matrix Formation and Cardiac Tissue Integrity

**DOI:** 10.1371/journal.pgen.1003616

**Published:** 2013-07-11

**Authors:** Maik Drechsler, Ariane C. Schmidt, Heiko Meyer, Achim Paululat

**Affiliations:** University of Osnabrueck, Department of Zoology and Developmental Biology, Osnabrueck, Germany; Harvard Medical School, Howard Hughes Medical Institute, United States of America

## Abstract

Here we report on the identification and functional characterization of the ADAMTS-like homolog *lonely heart* (*loh*) in *Drosophila melanogaster*. Loh displays all hallmarks of ADAMTSL proteins including several thrombospondin type 1 repeats (TSR1), and acts in concert with the collagen Pericardin (Prc). Loss of either *loh* or *prc* causes progressive cardiac damage peaking in the abolishment of heart function. We show that both proteins are integral components of the cardiac ECM mediating cellular adhesion between the cardiac tube and the pericardial cells. Loss of ECM integrity leads to an altered myo-fibrillar organization in cardiac cells massively influencing heart beat pattern. We show evidence that Loh acts as a secreted receptor for Prc and works as a crucial determinant to allow the formation of a cell and tissue specific ECM, while it does not influence the accumulation of other matrix proteins like Nidogen or Perlecan. Our findings demonstrate that the function of ADAMTS-like proteins is conserved throughout evolution and reveal a previously unknown interaction of these proteins with collagens.

## Author Summary

Cellular adhesion and tissue integrity in multicellular organisms strongly depend on the molecular network of the extracellular matrix (ECM). The number, topology and function of ECM molecules are highly diverse in different species, or even in single matrices in one organism. In our study we focus on the protein class of ADAMTS-like proteins. We identified Lonely heart (Loh) a member of this protein family and describe its function using the cardiac system of *Drosophila melanogaster* as model. Loh constitutes a secreted protein that resides in the ECM of heart cells and mediates the adhesion between different cell types - the pericadial cells and the cardiomyocytes. Lack of Loh function induces the dissociation of these cells and consequently leads to a breakdown of heart function. We found evidence that the major function of Loh is to recruit the collagen Pericardin (Prc) to the ECM of the cells and allow the proper organization of Prc into a reticular matrix. Since the function of Loh homologous proteins in other systems is rather elusive, this work provides new important insights into the biology of cell adhesion, matrix formation and indicates that ADAMTS-like proteins might facilitate an evolutionary conserved function.

## Introduction

The establishment and maintenance of extracellular matrices (ECM) are important tasks to allow proper organ function in metazoans. Among other factors, changes in ECM composition, turnover and homeostasis are crucial mediators of human cardiovascular disease leading to life threatening conditions and premature death. The ECM allows cells to resist mechanical forces, protects complex tissues from being damaged and promotes specific physical properties like elasticity or stiffness in order to maintain organ functionality. While the composition of the ECM is very complex and extremely variable the basic structural constituents can be grouped as collagens, glycoproteins and proteoglycans, which are highly conserved throughout metazoan species [1]. Consequently, defects in ECM proteins or matrix composition cause major developmental defects and strongly contribute to prevalent human disease like fibroses or cancer [2]. During the last years fibrotic disease and mutations in various ECM proteins were correlated to cardiovascular disease. For example mutations in human Col4a1 cause the weakening of the major vasculature leading to life threatening aneurysms or stroke [3] while mutations in murine Col4a1 and Col4a2 induce vascular defects causing internal bleedings and prenatal lethality [4]. Even more recently ADAMTS-like (ADAMTSL, A Disintegrin and Metalloprotease with Thrombospondin repeats) proteins have gained significant importance in the understanding of certain types of fibrillinopathies [5], [6]. Mutations in human ADAMTSL4 were identified in patients suffering from isolated ectopia lentis (EL), a recessive disorder of the occular lense [7], [8] and, more severely, aberrations in ADAMTSL2 cause geleophysic dysplasia a syndrome which, amongst others, manifests in the thickening of the vascular valves and progressive cardiac failure causing premature death [9]. Unfortunately, despite the pathological mutations no ADAMTSL alleles in genetically treatable model systems were described so far.

In the present study we use *Drosophila melanogaster* as a model of ECM function in the cardiac system. In *Drosophila* the maintenance of cardiac integrity is of great importance, since no mechanisms of cardiac cell replacement or tissue repair exist. A variety of mutations in ECM genes have been analyzed with respect to their function in different tissues and processes like neurogenesis, muscle attachment, wing development and others [10]–[12]. Cardiogenesis in the fly embryo depends on several ECM components including the evolutionarily conserved toolkit of proteins forming the basement membrane. The basement membrane constitutes a specialized type of ECM consisting of Laminins, Collagen IV, Perlecan and Nidogen found at the basal side of epithelial cells [13]. The interaction of laminins with cellular receptors like integrins or dystroglycan and its self-assembly into a higher meshwork forms the initial step of basement membrane formation in animals [14], [15]. Consequently, mutations in any of the four laminin encoding genes in *Drosophila* lead to severe embryonic cardiac defects. For example loss of *lanB1*, encoding the only β-subunit of the laminin trimer, prevents the accumulation of collagen IV and perlecan towards cardiac cells, while mutations in *lanA* and *lanB2* (encoding the α3,5-subunit and the γ-subunit, respectively) cause the detachment of pericardial cells, a specific type of nephrocytes in arthropods, from the heart tube [14], [16]–[18]. The highly abundant proteins forming the basement membrane have in common that they are distributed ubiquitously and cover all internal organs of the fly [14], [19].

Compared to that the cardiac ECM is unique, since it contains the collagen Pericardin (Prc), which is rather specifically decorating the heart tube [20], [21]. Prc displays certain homologies to mammalian collagen IV and was shown to be crucial for heart morphogenesis and cardiac cell to pericardial cell adhesion [20], [22]. However, the question of how Prc accumulates in a cell specific manner in the fly embryo or how specific matrices are specified in the rather open body cavity of insects in general was not addressed in detail so far. Here we introduce the gene *lonely heart* (*loh*), which is crucial to maintain cardiac integrity during postembryonic developmental stages. We show that Loh is a member of the ADAMTSL protein family and constitutes the essential mediator of Prc accumulation and matrix formation already in embryonic cardiac tissue. ADAMTSL proteins belong to the evolutionary conserved family of ADAM proteases with the exception that these proteins lack a proteolytically active domain in their primary sequence and therefore its function is unclear [5], [6]. We found evidence that Loh is sufficient to specifically recruit Prc to the ECM of different tissues indicating that Loh regulates the assembly of tissue and organ specific matrices. This is of great interest since the composition of the ECM determines its mechanical properties crucial for correct organ function and cellular behavior [23]. We also address the physiological relevance of cardiac integrity and show that lack of either *loh* or *prc* prevents proper blood circulation in the animals and cause a reduction of the fly's life span. The findings presented in here demonstrate that mutations in ADAMTSL proteins lead, like in human disease, to progressive heart failure and premature death in flies, strongly arguing for an evolutionary conserved function.

## Results

### Isolation of novel heart integrity mutants

**Figure 1:**
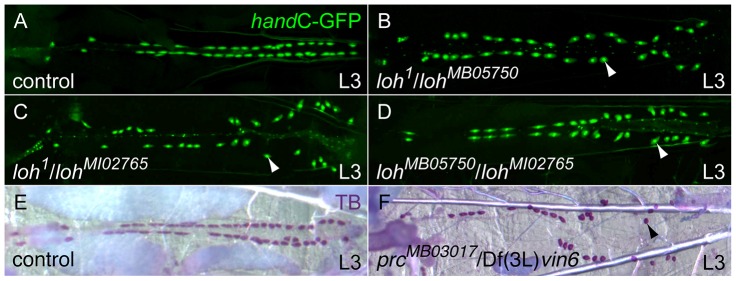
Isolation of new heart integrity mutants. (**A–D**) Combinations of *loh* mutant alleles, cardiac cells are marked with *hand*C-GFP. All transheterozygous mutant larvae display the detachment of pericardial cells (arrowheads) from the heart tube. (**E, F**) Pericardial cell detachment in transheterozygous *prc* mutant larvae (arrowhead in F). Pericardial cells were stained using toluidine blue (TB).

In order to identify novel mediators of cardiac function we screened a set of pupal lethal EMS induced mutants, known as the Zuker collection, for the presence of postembryonic cardiac malformations [24]. To mark all cells contributing to the mature heart we introduced the previously described *hand*C-GFP reporter into each individual mutant strain [25]. We identified a single allele, *lonely heart* (*loh^1^*), showing a strong detachment of pericardial cells from the heart tube during larval stages (Figure 1 and Figure S1A, B). To map the mutation to the genome we introduced the *loh^1^* allele to a collection of genomic deficiencies and assayed the progeny for the presence of the pericardial cell detachment phenotype. The allele failed to complement the deficiencies Df(2L)Exel7048, Df(2L)BSC453 and Df(2L)BSC144 but complements Df(2L)BSC209 (Figure S1C–F). This allowed us to narrow down the location of the mutation to a 14 kb genomic region at band 31E3-4 containing three open reading frames (Figure S1I). Since EMS is known to promote secondary hits on the same chromosome we decided to assay existing alleles of these three genes for the presence of the pericardial cell detachment phenotype. We were able to identify two alleles, MB05750 and MI02765, that are allelic to *loh^1^* and Df(2L)Exel7048 and produce the heart phenotype in transheterozygous condition (Figure 1A–D and Figure S1G, H). Both mutations were induced by the insertion of *minos* elements within the locus of the previously uncharacterized gene CG6232 [26], [27]. Based on sequence predictions CG6232 encodes an ADAMTS-like (A Disintegrin and Metalloproteinase with Thrombospondin repeats) protein, containing several Thrombospondin type 1 repeats, a central ADAM-spacer domain and a C-terminal Protease and Lacunin (PLAC) domain (Figure S1J). The primary sequence of Loh/CG6232 shows high homologies to mammalian ADAMTSL6, known to promote the formation of fibrillar matrices in mice [28].

**Figure 2:**
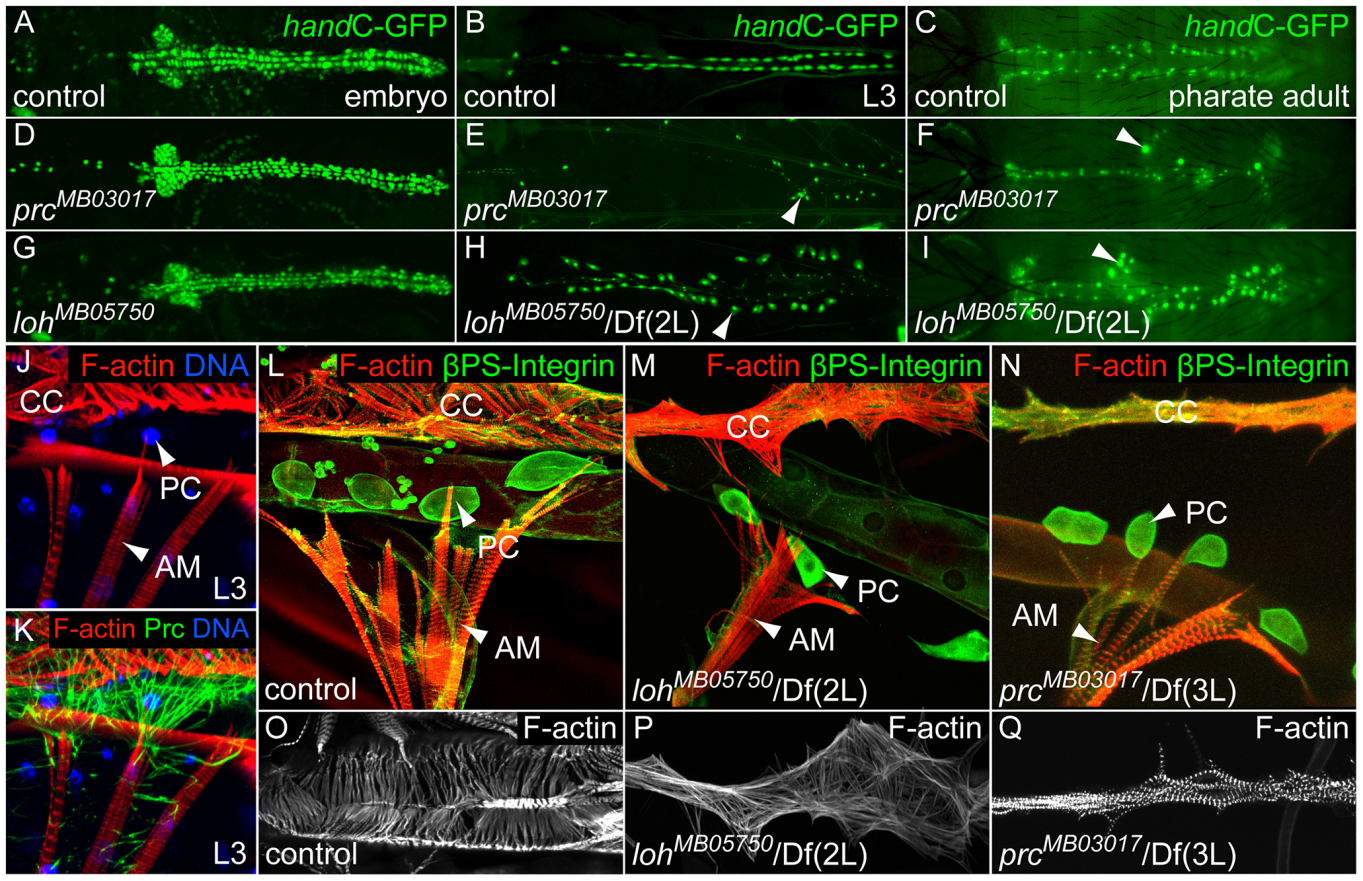
*loh* and *prc* are essential for cellular adhesion of cardiac cells. (**A–I**) Progressive loss of pericardial cell adhesion (cells marked by *hand*C-GFP) in *prc^MB03017^* (D–F) and *loh^MB05750^*/Df(2L)Exel7048 (G–I) mutants indicated by pericardial cell detachment (arrow heads). (**J, K**) Connection between the larval alary muscle (AM) and the cardiomyocytes (CC) is mainly facilitated by a reticular Prc matrix, covering the pericardial cells (PC). (**L–N**) Loss of cell adhesion includes the detachment of alary muscles (AM) from the heart in *loh^MB05750^*/Df(2L)Exel7048 (M) or *prc^MB03017^*/Df(3L)vin6 (N) third instar larvae. (**O–Q**) F-actin arrangement in control (O) *loh^MB05750^*/Df(2L)Exel7048 (P) and *prc^MB03017^*/Df(3L)*vin6* (Q) mutants third instar larvae. The orientation of the actin fibers is altered by loss of pericardial cell adhesion.

During a parallel reverse genetic approach we also tested transposon induced alleles affecting known ECM genes for the appearance of late cardiac defects. We identified the allele MB03017 carrying a *minos* element in the *pericardin* (*prc*) locus. Homozygous *prc^MB03017^* and transheterozygous *prc^MB03017^*/Df(3L)*vin6* animals display a strong pericardial cell detachment phenotype similar the *loh* phenotype (Figure 1E, F and Figure 2E). The Prc protein constitutes a rather heart specific collagen, which shows homologies to vertebrate collagen IV [22]. Previous studies implicated Prc to be involved in dorsal closure as well as cardiogenesis [20]. However, no gene specific mutant was available so far.

### *Lonely heart* and *pericardin* are essential for cardiac integrity but not cardiogenesis

To investigate the adhesion defects arising in both *loh* and *prc* mutants in more detail we analyzed the morphology of the heart at different developmental stages. During embryogenesis the heart tube arises from two bilateral primordia and forms a simple tube at the dorsal midline. Determination and migration of heart precursor cells is not affected in either *loh^MB05750^*/Df(2L)Exel7048 or *prc^MB03017^/prc^MB03017^* mutant animals (Figure 2A, D and G). During larval development the pericardial cells irreversibly detach from the heart tube with the phenotype becoming fully visible in third instar larvae (Figure 2B, E and H). The loss of cardiac integrity in both mutants does not constrain the development into adult animals and we could detect the pericardial cell detachment phenotype in pharate adult animals, which further develop into viable and fertile flies (Figure 2C, F and I). These findings show that the phenotype arises progressively during development and indicate that proper heart function is not essential for development into the imago. Of note the alleles *loh^1^* and *loh^MI02765^* cause larval lethality in homozygous condition, while the alleles are viable in transheterozygous combination indicating second site mutations or yet unknown dominant effects of the mutated proteins. Since *loh^MB05750^* and *prc^MB03017^* animals are homozygous viable and show the pericardial cell detachment phenotype all experiments predominantly focus on these two alleles.

Postembryonic pericardial cells are enclosed by a dense network of Prc fibers and connected to the alary muscles (Figure 2J–K). Since the heart tube and the alary muscles are not connected via direct cell-to-cell contacts this Prc network is likely to be a fundamental structural component to suspend the heart to the body cavity [29]. To evaluate the adhesion of the heart tube to the alary muscles in more detail we stained transheterozygous *loh^MB05750^*/Df(2L)Exel7048 and *prc^MB03017^*/Df(3L)*vin6* larvae for F-actin and βPS integrin (Figure 2L–N). The detachment of pericardial cells also ruptures the connection between the alary muscles and cardiomyocytes demonstrating that the lack of pericardial cell adhesion consequently lead to a breakdown of the heart's suspension towards the epidermis. Furthermore, the morphology of the cardiomyocytes itself is dramatically altered in *loh^MB05750^*/Df(2L)Exel7048 and *prc^MB03017^*/Df(3L)*vin6* mutants (Figure 2O–Q). While in the wild type cardiomyocytes show a defined arrangement of F-actin fibers in a circular fashion mutant cells exhibit an uncoordinated distribution of actin fibers and an altered cell shape. Since the arrangement of actin fibers might be a secondary effect of a changed cardiac cell polarity we stained mutant embryos for the polarity markers FasIII and αSpectrin (Figure S2A–L). Neither *loh* nor *prc* mutant hearts displayed changes in cell polarity proving that the changed actin arrangement is an effect of the defective cellular adhesion.

### The loss of cardiac integrity constrains circulatory activity

We next elucidated how heart beat is influenced in the mutants. For this purpose the beating pattern of the heart was recorded in semi-dissected third instar larvae (Movies S1, S2, S3) [30]. Wild type heart beat follows a very regular pattern and the heart walls display systolic and diastolic movements (Movie S1). Compared to that the beating pattern in *loh^MB05750^*/Df(2L)Exel7048 and *prc^MB03017^*/Df(3L)*vin6* mutant larvae is dramatically altered. The disorganized actin fibers cause a changed contraction movement of the whole organ along the posterior-anterior axis (Movie S2 and Movie S3). In addition no systole and diastole are detectable already indicating that the pumping performance of the organ is altered.

**Figure 3:**
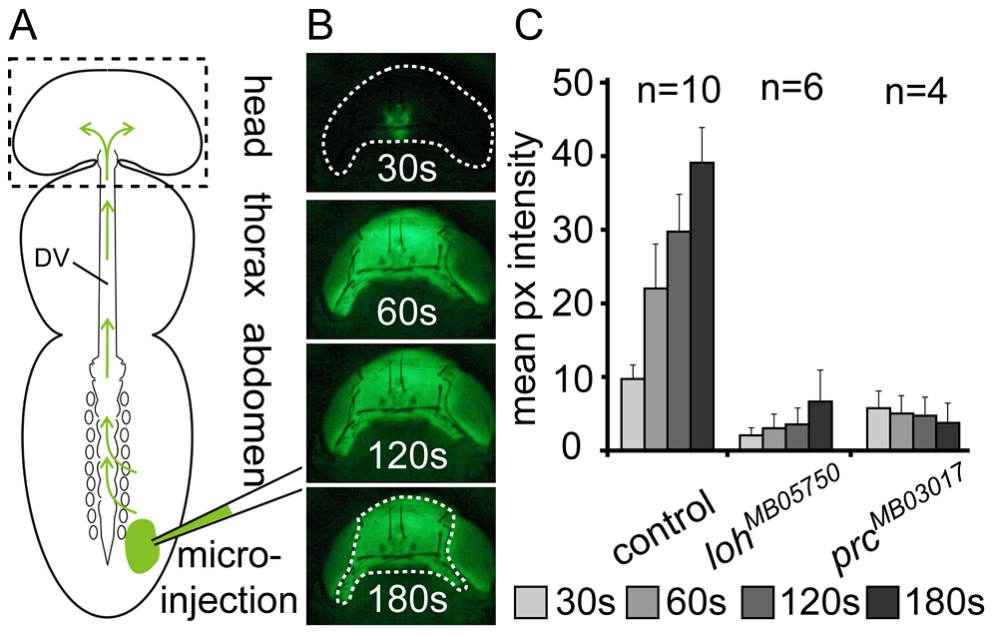
Loss of pericardial cell adhesion causes loss of heart function. (**A**) Scheme depicting the basics of dye angiography in pharate adults. The main body parts, the dorsal vessel (DV) and the injection area are indicated. (**B**) Head of a wild type animal (corresponding to dashed box in scheme A) showing the accumulation of the tracer at four consecutive time points after injection. (**C**) Mean pixel intensities measured at four consecutive time points showing cardiac output in wild type (*white^1118^*) and homozygous *prc^MB03017^* and *loh^MB05750^* pharate adult animals. Error bars are s.e.m. The region used for measurement is indicated in the lowest panel in B.

To evaluate whether the disruption of heart architecture and the changed beating pattern impairs heart functionality we analyzed the capability of mutant hearts to provide circulatory activity. To visualize the hemolymph flow by dye angiography we injected a fluorescent tracer into the abdomen of adult animals shortly before eclosion (pharate adults) and semi-quantified the pumping capacity of the dorsal vessel by measuring the tracer accumulation within the head (Figure 3A–C) [31]. To verify the reliability of the technique a control strain that does not display any cardiac defects was tested and showed a strong accumulation of the tracer in the head (Figure 3B–C and Movie S4). In contrast homozygous *prc^MB03017^* and *loh^MB05750^* mutant animals displayed a dramatic reduction or total absence of dye accumulation within the examination time, which proves that the observed disruption of heart integrity directly influences the ability to promote circulatory activity (Figure 3C).

Since it is known that heart failure can cause a significant reduction of *Drosophila's* life span [32], [33] we tested whether the isolated alleles show a direct effect on adult survival. As a wild type control we used the *white^1118^* strain, because this genotype resembles the genetic background of both *minos* insertion strains. Wild type flies (*white^1118^*) revealed an average life time of 46 days, while the mean life span of homozygous *loh^MB05750^* and *prc^MB03017^* animals was decreased by 26% (34 days) or 46% (25 days), respectively (Figure S3). This strongly argues that impaired cardiac function in the mutants reduces the survival of the animals.

### Molecular characterization of *loh* and *prc*

**Figure 4:**
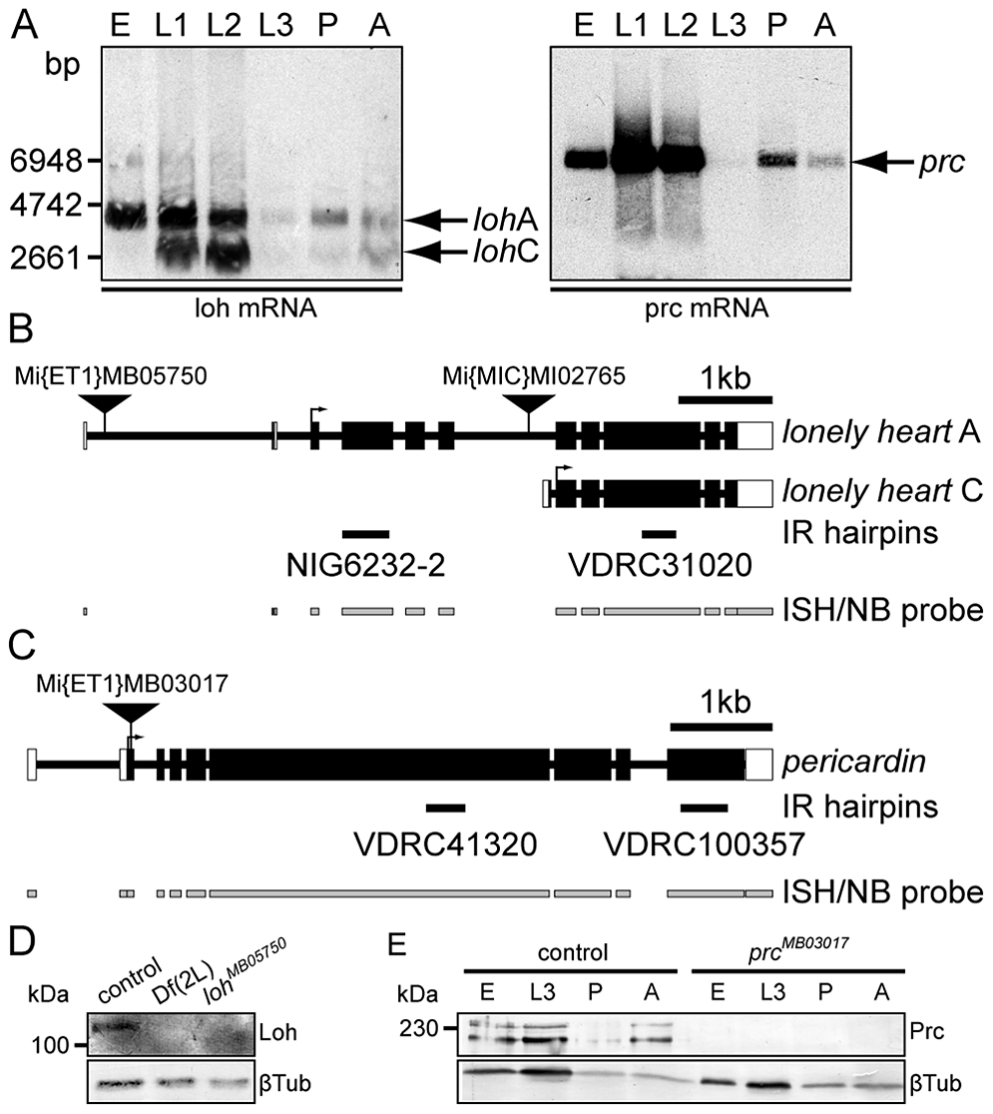
Molecular characterization of *loh* and *prc*. (**A**) Developmental Northern blots showing *loh* and *prc* expression in total RNA samples of 0–24 h old embryos (E), first, second or third instar larvae (L1–L3), mid-stage pupae (P) or adults (A) using gene specific riboprobes (indicated in B and C). (**B, C**) Schematic representation of *loh* (B) and *prc* (C) gene loci and transcripts. The schemes indicate the position of transposons, location of hairpins (IR) used for knock down and riboprobes used for Northern analysis (NB) and *in situ* hybridization (ISH). (**D**) Immunoblot of total protein extracts obtained from stage 17 control, homozygous Df(2L)Exel7048 or homozygous *loh^MB05750^* embryos probed with antibodies against Loh or βTub. Loh is undetectable in homozygous deficiency or mutant extracts. (**E**) Immunoblot of total protein extracts obtained from control or homozygous *prc^MB03017^* 0–24 h old embryos (E), third instar larvae (L3) mid-stage pupae (P) or adults (A) probed with antibodies against Prc or βTub. Prc is undetectable in extracts of homozygous mutants.

We investigated the temporal expression pattern of *loh* and *prc* by developmental Northern blots. The *loh* locus encodes two transcripts - a longer isoform A (3081 bp predicted) and a shorter isoform C (2131 bp predicted) (Figure 4A). While isoform A constitutes the major transcript during embryogenesis, isoform C becomes additionally expressed during the first and second larval stage (L1 and L2). Later on expression declines and becomes weakly re-activated during pupal and adult stages. Compared to *loh* the temporal expression profile of *prc* was found to be remarkably similar (Figure 4A). A single transcript (5535 bp predicted) becomes expressed from the embryo to L2 and declines in L3. During metamorphosis expression re-initiates and lasts until adulthood. In order to reveal if both *loh* isoforms are essentially needed to ensure proper heart integrity we expressed two independent gene specific hairpins either effecting only isoform A (*loh*-IR^NIG6232-2^) or both isoforms (*loh*-IR^VDRC31020^) under the control of *hand*C-Gal4 to knock down the gene's expression (Figure 4B). Expression of both hairpins causes a pericardial cell detachment phenotype. However, since expression of the *loh*-IR^NIG6232-2^ hairpin, which only targets isoform A, resulted in a detachment phenotype (Figure S4) we concluded that isoform A constitutes the relevant one for the observed adhesion defect.

To investigate the effect of the isolated mutations on the expression level we analyzed the total protein amounts by immunoblotting (Figure 4D, E). Therefore we raised a specific peptide antibody recognizing both Loh isoforms. In embryonic extracts the antibody detects a single protein band corresponding to isoform A. The band runs slightly higher compared to the predicted molecular mass of 100 kDa, most likely due to posttranslational modifications (Figure 4D). The protein is absent from extracts of homozygous Df(2L)Exel7048 embryos proving the specificity of the antibody. Significantly, the protein is also undetectable in extracts of homozygous *loh^MB05750^* embryos. RT-PCR analysis proved that *loh*A transcripts are severely reduced but not absent in these animals (Figure S4A), obviously leading to massively decreased protein levels. Similarly, Prc protein could be detected in extracts of different developmental stages in the control, but is absent from homozygous mutants (Figure 4E).

### *Lonely heart* and *pericardin* show similar spatial expression patterns

**Figure 5:**
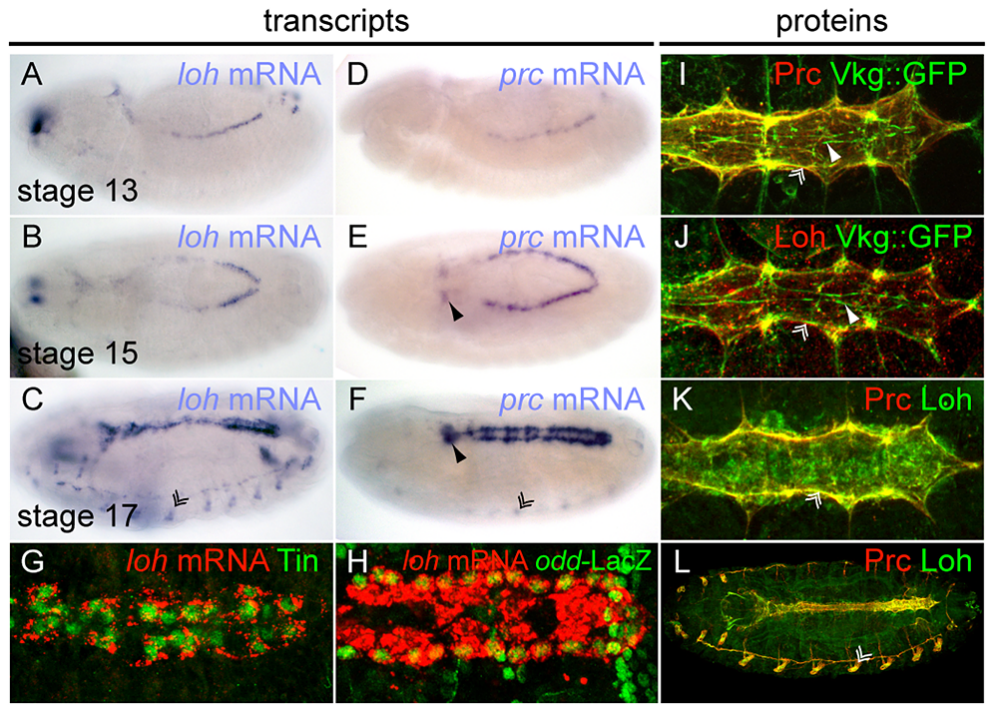
Embryonic expression and localization of Loh and Prc. (**A–F**) Whole mount *in situ* hybridization revealing the expression patterns of *loh* (A–C) and *prc* (D–F) during embryogenesis. Chordotonal organs (double arrowhead in C), the ring gland (arrowhead in E, F) and oenocytes (double arrowhead in F) are indicated. (**G, H**) Double labeling of *loh* transcripts and Tinman (G) or *odd*-LacZ (H) in stage 17 embryos. (**I, J**) Co-staining of either Prc (I) or Loh (J) with Vkg::GFP in stage 17 embryos demonstrates localization within the cardiac ECM at the basal side of cardiomyocytes. (**K, L**) Co-staining of Loh and Prc shows co-localization of both proteins.

Given the similar phenotypes of the mutants we sought to analyze the spatial expression pattern of both genes. Transcripts of *loh* and *prc* can be detected from embryonic stage 13 onwards until the end of embryogenesis in cardioblast and pericardial cell precursors (Figure 5A–F), where *loh* seems to be more prominently expressed in the ventricle of late stage embryos (Figure 5C). Additionally, *loh* transcripts were detected in the chordotonal organs, while *prc* is expressed by the oenocytes. Since it is known that *prc* is only expressed by a subset of cardiac cells we analyzed the expression of *loh* mRNA in combination with the cardiac cell markers Tinman and *odd skipped*-lacZ [20], [34], [35]. *loh* transcripts are expressed by both cell types demonstrating that most cardioblasts and pericardial cells contribute to the gene's expression (Figure 5G, H). As previously reported, Prc protein distributes predominantly along the basal side of the cardiomyocytes where it co-localizes with the collagen IV fusion protein Vkg::GFP (Figure 5I) [20], [36]. Strikingly, Loh co-localizes with Vkg::GFP as well as Prc, demonstrating that it constitutes an integral part of the basal cardiac ECM (Figure 5J–K). The detected signal was considered to be specific since it follows the observed mRNA pattern and is undetectable in homozygous Df(2L)Exel7048 embryos (Figure S5A–C). The expression of Loh and Prc supports a function in mediating the adhesion between pericardial cells and cardiomyocytes in the mature heart, while the observed co-localization throughout the whole embryo indicates a cooperative function (Figure 5L).

### The extracellular localization of Prc depends on Loh but not vice versa

**Figure 6:**
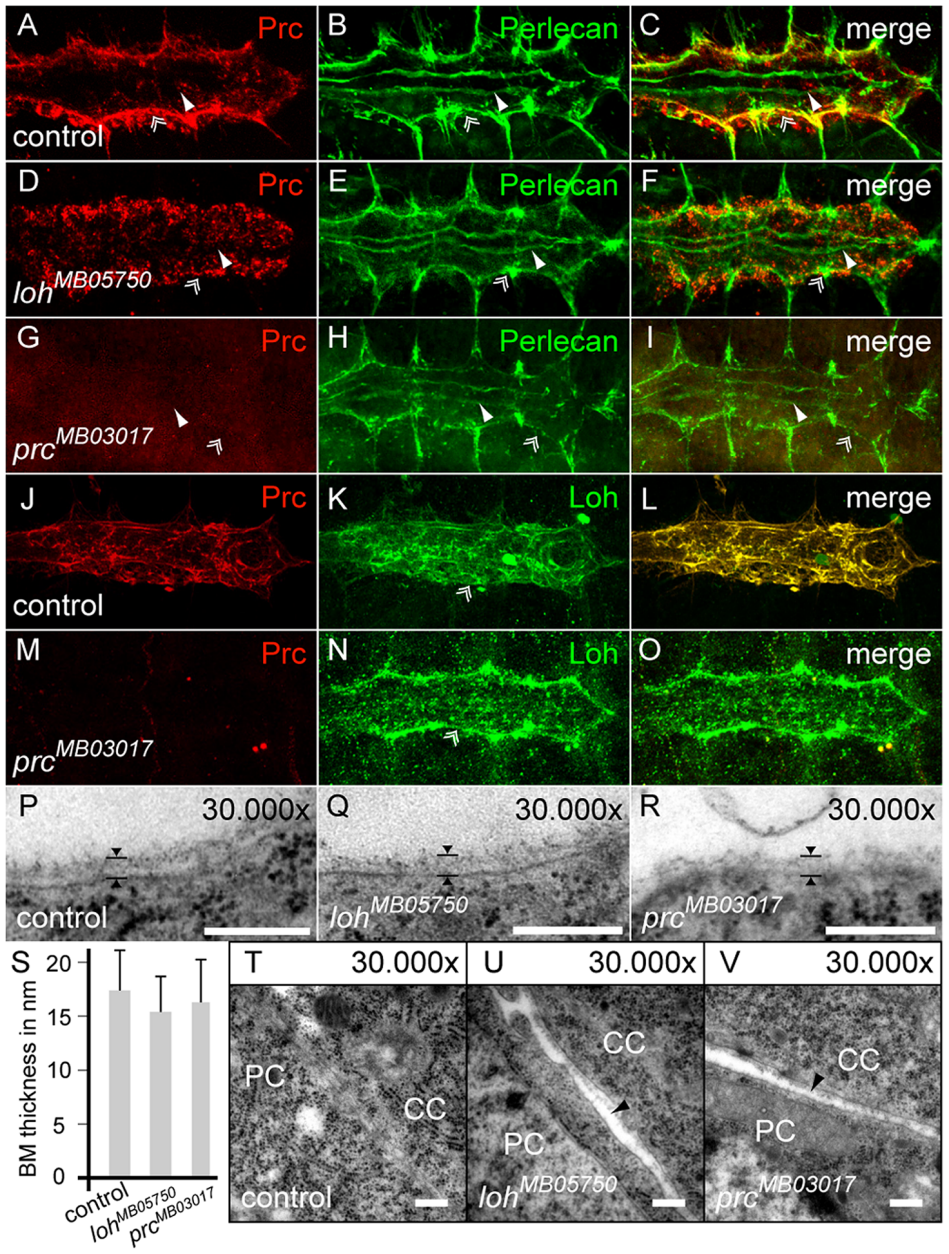
Localization of Prc depends on Loh but not vice versa. (**A–I**) Localization of Perlecan and Prc in stage 17 control embryos (A–C), compared to homozygous *loh^MB05750^* (D–F) or homozygous *prc^MB03017^* mutants (G–I). Prc but not Perlecan becomes mis-localized by the absence of Loh. (**J–O**) Localization of Loh in stage 17 control embryos (J–L) compared to homozygous *prc^MB03017^* mutants (M–O). The localization of Loh to the ECM is not affected by the absence of Prc. Of note, the anti-Loh antiserum needs heat fixation leading to a different appearance of Prc in the stained control animals compared to chemical fixation as shown in A. (**P–R**) The luminal ECM of cardiomyocytes at embryonic stage 17 is not altered in either homozygous *loh^MB05750^* or *prc^MB03017^* mutants. The arrowheads indicate the thickness of the ECM. (**S**) Quantification of luminal basement membrane (BM) thickness in animals of the indicated genotypes. Mutants do not show significant alterations in ECM thickness. Error bars shown are standard deviation (s.d.) (**T–V**) TEM section of the adhesion area between cardiomyocytes (CC) and pericardial cells (PC). Lack of either *loh* or *prc* cause gaps between the cells. Scale bars are 250 nm.

The data presented so far pointed us to the question if Loh and Prc act cooperatively in the cardiac ECM. To test if the proteins affect each other we analyzed the localization of Prc in *loh* mutant background and vice versa (Figure 6A–O). In homozygous *loh^MB05750^*, *loh^MI02765^* and *loh^1^* embryos Prc becomes normally secreted but strikingly fails to assemble properly in between the pericardial cells and the heart (Figure 6A–F and Figure S6A, B). While in the wild type Prc organizes into a proteogenic sheet at the basal side of the cardiomyocytes this regular distribution is completely disrupted in *loh* mutant embryos (Figure 6A–F). We also tested whether impaired *loh* expression affects other ECM proteins like Laminin, Nidogen or Perlecan (Figure 6B, E and Figure S6F, G and I,J). The expression and distribution of all tested proteins was unchanged in *loh* mutant animals indicating that Loh specifically regulates the correct accumulation of Prc but is not needed for ECM formation in general. The other way around the lack of Prc in homozygous *prc^MB03017^* embryos does not affect the localization of Loh (Figure 6J–O) or any other tested ECM protein demonstrating that the function of both proteins is not mutual (Figure 6G–N and Figure S6H, K). To prove that the phenotypes in *loh^MB05750^* and *prc^MB03017^* definitely arise from the inserted transposons we generated revertants by precise excision of the *minos* elements [26], which was verified by PCR and subsequent sequencing (Figure S6C–E). The precise remobilization of both transposons lead to a restored Prc expression and distribution in both revertants demonstrating that the mutations are gene specific.

To study the effect of *loh* and *prc* mutants on heart cell morphology in more detail we investigated TEM cross sections of wild type and homozygous *loh^MB05750^* and *prc^MB03017^* embryos (Figure 6P–V and Figure S6L–N). Like in wild type the cardiomyocytes are localized along the dorsal midline at the end of embryogenesis in both mutants showing that dorsal closure is not affected (Figure S6L–N). However, frequently the cardiomyocytes in homozygous *prc^MB03017^* mutants fail to seal the lumen properly at the ventral side of the heart tube (Figure S6N). Staining against the ligand Slit, which is involved in heart lumen formation did not reveal any changes in its distribution indicating that the Slit/Robo signaling cascade is not affected (Figure S6O–Q) [37]. Most importantly, the luminal and basal membranes of the cardiomyocytes are covered by a distinct basement membrane in both homozygous mutants supporting the immunocytochemical data (Figure 6P–R). Measuring its thickness does not reveal any significant changes (Figure 6S). However, even if the pericardial cells are not fully detached from the embryonic heart, small gaps between the cells and rupture of the connecting ECM are detectable (Figure 6T–V). Taken together these data demonstrate that Prc and Loh are essential to maintain pericardial cell to cardiomyocyte adhesion and heart integrity but are not involved in ECM formation in general.

### Prc becomes secreted by the larval fat body and recruited to the cardiac ECM

**Figure 7:**
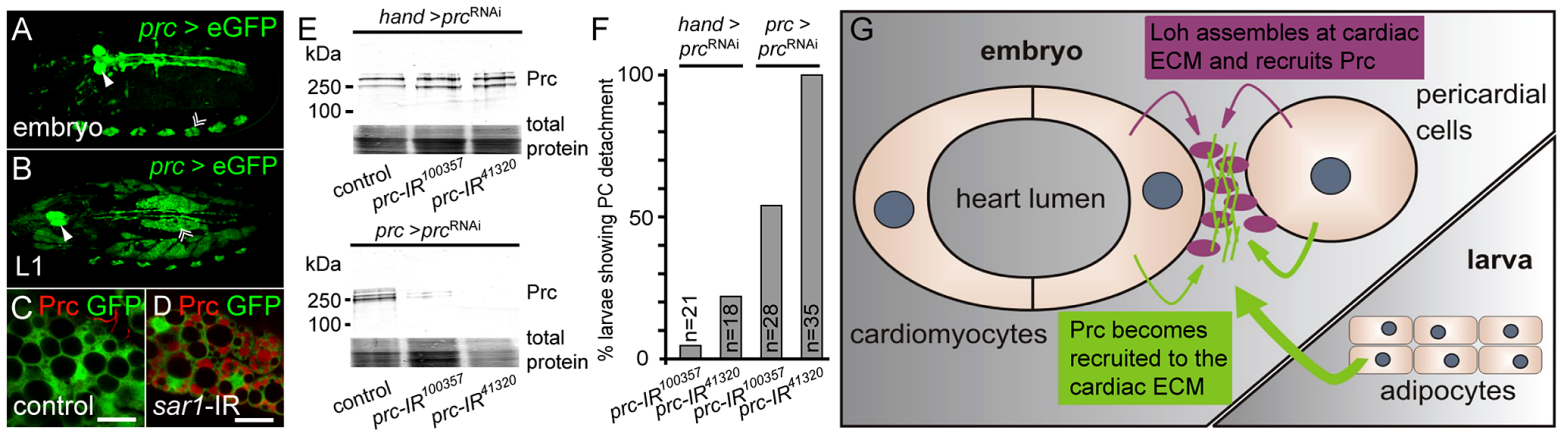
Prc becomes secreted by the larval fat body and recruited to the heart. (**A, B**) Embryonic (stage 17) and larval (L1) expression pattern of the *prc*>GFP reporter. (**C, D**) Prc protein becomes trapped in adipocytes of *prc*>*sar1*-IR knock down first instar larvae, proving that the protein becomes expressed by the fat body. Scale bar is 10 µm. (**E**) Immunoblots of whole larval extracts probed against Prc. While *hand*C-Gal4 driven knock down does not alter the total amount of Prc the protein is nearly undetectable in *prc*-Gal4 driven knock down animals. Total protein was stained with amido black 10B. (**F**) Percentage of third instar larvae showing pericardial cell detachment induced by *prc* knock down either driven by *hand*C-Gal4 or *prc*-Gal4. *n* indicates the total number of tested animals. (**G**) Scheme of Prc matrix formation. Secreted Prc from different sources (heart cells in the embryo, adipocytes in the larva) becomes incorporated into the cardiac ECM dependent on Loh.

Hypothetically the open circulatory system of insects would allow ECM proteins to be expressed by a certain cell type, then be distributed over the blood flow and finally become recruited by specific receptors expressed on the target cells. The embryonic expression pattern of *loh* and *prc* argue that both proteins are primarily produced locally by heart cells and become secreted into the cardiac ECM. To analyze the expression of *prc* during later stages we used the previously described *prc*-Gal4 driver to express GFP and found that it exactly mimics the expression pattern of *prc* in the embryo (Figure 7A) [20]. Upon larval hatching the driver becomes strongly activated in the fat body (Figure 7B) raising the question, whether the reporter mimics the endogenous *prc* expression. To test if Prc becomes produced by adipocytes we trapped the protein by inhibiting the protein secretion machinery of the cell by knocking down the expression of the small GTPase Sar1, which is essential for the establishment of COPII coated vesicles and protein secretion (Figure 7C, D) [38]. Compared to wild type, adipocytes of *prc*>*sar1*-IR first instar larvae displayed a strong accumulation of intracellular Prc protein unambiguously demonstrating that it becomes expressed by the larval fat body. To estimate the contribution of fat body derived Prc to the total amount of the protein made, we knocked down *prc* expression either in heart cells alone (*hand*C-Gal4) or in both heart and fat body (*prc*-Gal4) and detected the protein by immunoblotting (Figure 7E). The specificity of the knock down was ensured by the use of two independent hairpins (Figure 3C). Prc levels are not markedly changed in *hand*C>*prc*-IR third instar larvae, while the protein is nearly undetectable in extracts of *prc*>*prc*-IR animals illustrating that most of the larval Prc protein becomes secreted by adipocytes. Finally, the pericardial cell detachment phenotype could be induced by knocking down *prc* expression using both drivers (Figure S7). However, the penetrance of the induced pericardial cell detachment phenotype is strikingly higher if the knock down was mediated via *prc*-Gal4 (Figure 7F), showing that the protein secreted from adipocytes indeed contributes to pericardial cell adhesion. From these experiments we conclude that the major source of Prc in larvae is non-cardiac tissue. Nevertheless, locally produced Prc contributes to proper heart integrity, since heart specific knock down of Prc expression does induce the detachment phenotype as well. Taken together these experiments prove a developmental switch in Prc expression with embryonic Prc being locally produced by cardiac cells and during later stages becoming mainly secreted by the fat body (Figure 7G). Furthermore, the integration of fat body derived Prc into the cardiac ECM is essential to promote organ integrity.

### Loh mediates the recruitment and formation of Prc matrices *in vivo*

**Figure 8:**
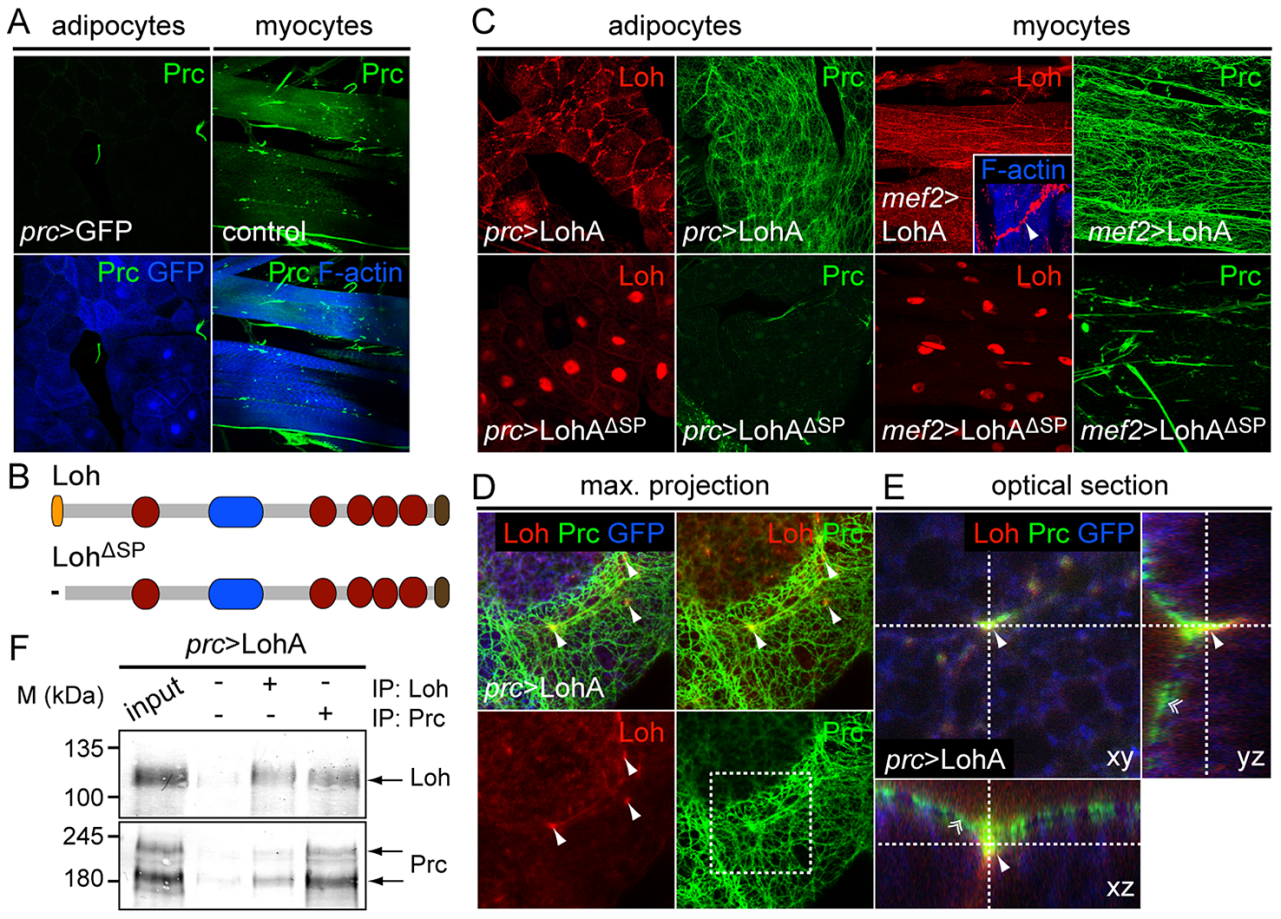
Loh induces Prc matrix formation *in vivo*. (**A**) Prc (green) is absent from the ECM of larval muscles or the fat body in wild type third instar larvae. (**B**) Schematic representation of ectopically expressed LohA wild type protein or the secretion defective variant LohA^ΔSP^. (**C**) Expression of LohA (red) in adipocytes or myocytes results in the formation of a dense Prc matrix (green) along both cell types in third instar larvae, while secretion of LohA is essential to mediate Prc recruitment. Inset shows accumulation of LohA at muscle attachment sites. (**D**) Loh and Prc partially co-localize in an artificial matrix around adipocytes. Loh distributes as weak fibers accumulating in a spotted fashion along cellular contacts (arrowheads). (**E**) Single optical slice of adipocytes. Loh co-localizes to Prc (arrowhead) at the root of Prc fibers (double arrowhead). (**F**) Loh co-immunoprecipitates with Prc and vice versa from total adult protein extracts demonstrating a biochemical interaction of both proteins.

Although Prc is produced by adipocytes, the protein is not incorporated into the ECM of the fat body indicating that these cells lack specific adhesion properties for Prc (Figure 8A). We found that in third instar larvae the protein almost exclusively accumulates around tissues that initially expressed *loh* during embryogenesis, but is nearly absent from other mesodermal tissues. From these observations we concluded that Loh might act as a mediator or receptor of Prc matrix formation in *Drosophila*. To test if Loh is indeed sufficient to induce the formation of Prc matrices we expressed the protein ectopically either in adipocytes or myocytes by using *prc*-Gal4 or *mef2*-Gal4, respectively. Even if some sole Prc fibers can be found along both cell types these organs are not naturally covered by a Prc matrix (Figure 8A). Ectopically expressed LohA protein becomes secreted from both cell types and localizes around the cells (Figure 8C). The protein is retained at the cell surface of adipocytes or myocytes indicating proper localization in the ECM. Upon expression in the fat body, LohA distributes along the whole organ showing a higher accumulation at cellular contacts. Similarly, LohA ectopically expressed by myocytes distributes along the whole myotube with higher accumulation at the muscle tendons (Figure 8C, inset). Most importantly, we found that LohA expression strongly induces the formation of an ectopic proteogenic Prc network around both cell types (Figure 8C). Adipocytes and myocytes ectopically expressing LohA are tightly covered by Prc fibers, which are interconnected to each other and form a dense meshwork. Immunoblot analysis on whole extracts revealed that the overall amount of Prc was not changed in these animals (Figure S8A), demonstrating that ectopic LohA expression leads to a re-direction of Prc protein. To evaluate if Loh acts within the ECM we ectopically expressed a secretion defective version of the protein (Figure 8B), lacking the N-terminal signal peptide. The mutated protein localizes to the nuclei of the cells and fails to recruit Prc to the target matrix demonstrating that LohA has to be secreted in order to act as an initiating factor of Prc matrix formation.

To evaluate if both proteins co-localize in such artificial matrices we counterstained dissected *prc*>LohA third instar larvae for Loh and Prc (Figure 8D,E). High resolution images of dissected fat bodies showed that ectopic LohA distributes as a very faint network at the surface of adipocytes and clusters in a pointy fashion along the cell contacts (Figure 8D) but does not completely co-localize with the recruited Prc fibers. Single slices and optical cross sections further demonstrate that Loh co-localizes with Prc at the anchoring points of the Prc network (Figure 8E), indicating that Loh might connect the root of each Prc fiber to the cell surface. Eventually, co-immunoprecipitation experiments using protein extracts isolated from *prc*>LohA adults proved a either direct or indirect biochemical interaction of both proteins (Figure 8F). In the respective experiments Prc co-precipitated if Loh was pulled down and vice versa. Based on these findings we hypothesize that Loh acts as a linker protein allowing Prc to interact with the cell surface, and wondered if Loh co-localizes with specific cell surface receptors. We found that LohA co-localizes to βPS integrin in adipocytes of *prc*>LohA third instar larvae (Figure S8B) tending us to speculate that LohA binds to integrin receptors, which has to be proven by further experiments. In summary we found that LohA is a crucial and sufficient mediator of Prc matrix formation, very likely acting by interconnecting Prc with the cell's ECM.

## Discussion

### The gene *lonely heart* is essential for cardiac matrix formation and stability

In this study we demonstrate that the *Drosophila* ADAMTSL protein Loh constitutes an unique protein of the cardiac ECM, essentially mediating cell adhesion and matrix formation. Loh is the first protein of its family identified and characterized in depth in flies. We isolated three independent alleles of the gene, all displaying the very same phenotype - the detachment of pericardial cells from the contracting heart tube during larval stages. Thus, the gene *loh* constitutes a novel and essential mediator of heart cell adhesion and cardiac function. Surprisingly, impaired heart function does not hamper proper development into adult animals but significantly reduces life span. This might be explained by the fact that oxygen transport and blood flow is uncoupled in insects and therefore a reduced hemolymph circulation might not immediately result in cytotoxicity. Furthermore, the open body cavity of the larvae might also allow a distribution of hemolymph independently of a pumping organ supporting the finding that larvae seem not to achieve any drawbacks by the loss of heart function.

Based on the primary sequence the domain architecture of Loh is extremely similar to that of vertebrate ADAMTSL6 and is likely to be its ortholog. Furthermore, ADAMTSL6 is the only protein of this family known to produce two transcriptional isoforms from one gene locus. In contrast to Loh the shorter ADAMTSL6 isoform was found to be functional in organizing the ECM in mice [28]. Our data demonstrate that LohA, the larger protein, is functional and sufficient to mediate matrix formation in *Drosophila* while the role of the shorter isoform C remains elusive by now. However, since the *lohC* transcript is not expressed during embryogenesis, the critical time window of *loh* function, we exclude any role of LohC in mediating cardiac ECM formation.

By testing different ECM proteins we demonstrated that Prc, a collagen with a very restricted distribution in the animal, is particularly affected in all isolated *loh* mutant alleles, emphasizing the specific function of Loh to promote Prc matrix formation. Consequently, we isolated the first *prc* mutant allele, which phenocopies the cardiac defects found in *loh* mutant strains. In *loh* mutant animals Prc mislocalizes along the heart already during embryogenesis, leading to a progressive loss of tissue integrity, which eventually causes the observed collapse of the heart tube and an abolishment of cardiac activity. The main function of both proteins is therefore the mediation of cellular adhesion between the heart, the pericardial cells and the alary muscles which further connect the whole organ system to the body cavity.

In addition to the cell adhesion defects we also found that the process of heart lumen formation was impaired in *prc* but not *loh* mutants. Since we have not followed up the details of this phenotype the role of Prc in lumen formation remains elusive for now. However, the data implicates that the presence of Prc is critical to allow cardioblasts to seal the lumen correctly, while the correct localization of Prc into the matrix seems not to be essential for this process.

### The cardiac matrix is established during embryogenesis and maintained in the larva

Analyzing the embryonic and larval expression patterns of *loh* and *prc* revealed that both genes are predominantly active during the growing stages of the animal and become deactivated after the heart has grown to its final size. In the embryo, both genes are transcribed in either the same or very proximate cells indicating that the proteins are not distributed over longer distances once they are secreted. Importantly, the final localization of Prc therefore mainly follows the expression of *loh*. This can be seen best in the oenocytes of the embryo, where Prc becomes secreted but later on mainly localizes to the overlying chordotonal organs that in turn express *loh*. Thus, *loh* expression is a prerequisite for the successful establishment of a Prc matrix. This local protein distribution changes during larval stages. As demonstrated by an inhibited secretion in adipocytes of *prc>sar1*-IR animals, Prc becomes strongly expressed by the fat body during early larval stages. Hence, the protein becomes distributed over longer distances in the larva but still decorates organs and tissues that initially expressed *loh*. Based on these data, we provide a conceptual model (Figure 7G) in which Loh predetermines the ECM to allow Prc to become coupled to the cell surface and to be organized into a reticular matrix. Previously it was shown that Collagen IV, the major collagen in the basement membrane, becomes also secreted by adipocytes and distributes through the hemolymph [39]. We can now prove that Prc as a second collagen is also synthesized by the larval fat body, which enhances the importance of this organ for ECM biogenesis. The developmental change in *prc* expression might therefore be explained by the ongoing differentiation of pericardial cells into mature nephrocytes during larval stages. While embryonic pericardial cells are able to secrete large amounts of protein into the extracellular space, the major function of pericardial nephrocytes is endocytosis [40], thus requiring adipocytes to take over Prc production. Finally our results show that the cardiac matrix is maintained during larval growing phases presumably by the consecutive incorporation of fat body derived Prc.

### Loh becomes incorporated into the ECM

The ectopic expression of Loh showed that the secreted protein is readily incorporated into different matrices raising the question how Loh itself interacts with the ECM in general. At the moment it is not fully understood if ADAMTSL proteins interact with miscellaneous ECM components or require specific cell surface receptors. Based on the spatial proximity of Loh to βPS integrin we speculate that Loh may interact with integrin receptors and link these to Prc bundles, thereby promoting the connection of the Prc network to the cell surface. This idea is supported by the observed changes in fiber orientation of mutant cardiomyocytes. Since it is known that integrins are connected to the underlying Z-disks of muscle cells by a structure called the costamere [41] we propose that lack of integrin-ECM binding induces the redistribution of myofibrils. However, there is no evidence of an interaction between ADAMTSL proteins and integrins or any other cellular receptor so far. Nevertheless, in such a model Loh would allow the specific binding of specialized ECM molecules to only some unique matrices. Since *Drosophila* possesses only two β integrin subunits the number of α/β-dimers is limited and the use of Loh as an adapter molecule increases the diversity of matrix composition and opens up the possibility to create sub-functional matrices. Furthermore, integrin mediated binding seems to influence the correct assembly of Prc since previous findings already showed that lack of αPS3- or βPS integrin can interfere with the distribution of Prc and induce pericardial cell detachment phenotypes [42].

In addition to a receptor mediated ECM incorporation of Loh, binding might also be achieved by some or all of the five TSR1 domains found in the primary sequence of the protein. Previously it was demonstrated that ADAMTS(L) proteins can bind to the ECM via the various TSR1 motifs that interact with glycosaminoglycans [43]. This would not need special receptors and allow Loh to incorporate into any matrix. The cell specific expression of *loh* would then mainly decide which matrix will incorporate Prc and this would in turn strongly depend on the *cis*-regulation of the gene's expression.

### Loh acts as receptor allowing matrix sub-functionalization

On the molecular level we propose that Loh basically acts as a linker protein. Based on the ectopic expression of Loh and the co-immunoprecipitation experiments we can demonstrate that Loh and Prc interact *in vivo*. In our hands Loh behaves like a secreted receptor molecule that specifically recruits Prc to the cell surface. Our findings indicate that the main molecular function might therefore be binding, but does not exclude additional functions of the protein. It was suggested previously that ADAMTSL proteins act as regulators of extracellular proteases and thereby regulate ECM content and composition [6]. For example it was demonstrated that *Drosophila* Papilin, another member of ADAMTSL related proteins, is sufficient to inhibit a vertebrate procollagen proteinase *in vitro* [44]. Thus, it is possible that also Loh regulates a so far unknown proteinase that renders the matrix unsuitable for the accumulation of Prc in some way. In such a model the activity of Loh would then influence the pre-existing microenvironment around a cell to allow Prc to assemble into a network. However, there is no evidence for such a function or the involvement of proteinases so far.

### ADAMTS-like proteins act as mediators of fibrillar matrices

The observed roles of Loh in *Drosophila* partially reflect the function of ADAMTSL proteins in vertebrates, which were shown to organize Fibrillin-1 (FBN1) microfibrils in specialized matrices. Genetic and biochemical analyses showed that ADAMTSL4 and ADAMSTL6 are sufficient to mediate the formation of FBN1 fibrils in cultured fibroblasts as well as *in vivo* [28], [45]. ADAMTSL4 acts as a FBN1 binding protein that mediates microfibril assembly in the zonule fibers of the human eye leading to isolated ectopia lentis (IEL) if mutated. Thus, IEL is caused predominantly by altered mechanical properties of the zonular fibers leading to a progressive dislocation of the lens [45]. In *Drosophila*, where no FBN1 homolog exists, Loh interacts with Prc and mediates its distribution within the ECM in a very similar manner. Therefore, the correct assembly of Prc between the pericardial cells and the heart tube could promote the mechanical properties needed to sustain the permanent mechanical forces during heartbeat. The clinical phenotypes of geleophysic dysplasia (GD) observed in ADAMTSL2 mutant patients exceed a function of simply promoting mechanical stability of the ECM. It was shown that ADAMTSL2 binds to FBN1 but also interacts with LTBP1, a regulator of TGFβ signaling, and therefore the phenotypes of GD also include growing defects, muscular hypertrophy and thickening of the skin [9]. None of these additional phenotypes were observed in *Drosophila loh* mutants. Therefore, it is obvious that ADAMTSL proteins developed novel functions during evolution making them essential mediators of ECM development and homeostasis. So far there are no reports of interactions between any ADAMTSL proteins with collagens but the obviously similar functions in flies and vertebrates strongly argue for a conserved function in organizing fibrillar matrix proteins.

## Materials and Methods

### *Drosophila* genetics

Flies were kept under standard conditions at 25°C on cornmeal agar. The following fly stocks were obtained from the Bloomington stock center: *w^1118^*; Mi(ET1)*prc^MB03017^*/TM6c,*Sb^1^*, *w^1118^*; Mi(ET1)*loh^MB05750^*, *y^1^*,*w^1118^*; Mi(MIC)*loh^MI02765^*/SM6a, Df(2L)Exel7048/CyO, Df(3L)*vin6*/TM3, *Sb^1^*,*Ser^1^*, *w^1118^*; Sco/SM6a,P{hsILMiT}2.4, *w^1118^*; UAS-eGFP and balancer stocks Kr*^If-1^*/CyO,*Kr*>GFP and *Dr^1^*/TM3,*Kr*>GFP.

Further fly stocks used are: *hand*C-GFP and *hand*C-Gal4 [25], *odd^rk111^* (*odd*-lacZ) (C. Rauskolb), *vkg*::GFP-454 [36], UAS *prc*-IR41320, UAS *prc*-IR100357, UAS *loh*-IR31020 and UAS *Sar1*-IR34191 [46], UAS *loh*-IR6232-2 (*Drosophila* Genetic Resource Center, Kyoto), *mef2*-Gal4 (H. Nguyen) and *prc*-Gal4 [20].

### Re-mobilization of *minos* elements

Precise excision of *minos* elements was carried out essentially as described before [26]. Briefly, homozygous *w^1118^*; Mi(ET1)*loh^MB05750^* or *w^1118^*; Mi(ET1)*prc^MB03017^* males were mated to *w^1118^*; Sco/SM6a, P{hsILMiT}2.4 “jump starter” females. After two days adults were removed and the F1 progeny was heat shocked each day at 37°C for 1 h until hatching. F1 males, carrying the *minos* element (expressing GFP) and the transposase source (recognized by the SM6a balancer) were mated to adequate balancer stocks. In the F2 generation revertant chromosomes were identified by the absence of GFP expression and isolated via backcrossing to the F1 balancer stocks. Revertant lines were established and removal of the *minos* elements was evaluated by amplifying closely flanking sequences of the transposon by PCR and sequencing. Oligonucleotides (*minos*-flank) used for PCR and sequencing are: *loh*-fwd GCGGTCAGCTAAATAGCATC, *loh*-rev GAATTGGTTTGTCCCACAACG, *prc*-fwd CACACAGTGGAGCGAGATCC and *prc*-rev CCTTTCGAAGTGTAAAGTGC.

### Immunohistochemistry

Embryos were prepared for staining by chemical or heat fixation as described previously [47], [48]. Staining of larvae was done on dissected tissue samples, fixed 1 h in 3.7% formaldehyde in 1× PBS. Primary antibodies used are: guinea pig anti-Loh (1∶500, heat fixation, this study), mouse anti-Prc/EC11 (1∶5, Developmental Studies Hybridoma Bank, DSHB), mouse anti-βPS integrin/CF.6G11 (1∶3, DSHB), mouse anti-FasIII/7G10 (1∶3, DSHB), mouse anti-αSpectrin/3A9 (1∶3, DSHB), mouse anti-Slit/C555.6D (1∶3, heat fixation, DSHB), rabbit anti-Perlecan/Trol (1∶1.000) [49], rabbit anti-Nidogen/Entactin (1∶1.000, a gift from S. Baumgartner), rabbit anti-Laminin (detects only secreted Laminin trimers; a gift from J. Fessler), rabbit anit-Tinman (1∶800) [34] and rabbit anti-GFP (1∶1.000, Abcam). Secondary antibodies used are anti-mouse-Cy2/Cy3 (1∶100/1∶200, Dianova), anti-rabbit-Cy2/Cy3 (1∶100/1∶200, Dianova) and anti-guinea pig-Cy2/Cy3/Alexa633 (1∶100/1∶200/1∶200, Dianova and Abcam). F-Actin was visualized by staining fixed tissues using TRITC coupled phalloidin (Sigma), at a concentration of 0.4 µg/ml in 1× PBS, for 1 h at room temperature. All images were acquired using a Zeiss LSM 5 PASCAL confocal microscope and standard objectives.

### Staining of nephrocytes using toluidine blue

The ability of insect nephrocytes to sequester colloids from solutions can be used to specifically label living cells. Therefore colloidal toluidine blue was used as vital stain. Third instar larvae were dissected in 1× PBS and incubated in 0,1 mg/ml colloidal toluidine blue solution for 1 min. Living nephrocytes specifically take up the dye resulting in a deep blue staining. Unspecific signals were removed by three consecutive washes in 1× PBS and animals were photographed immediately.

### Immunoblotting

Embryonic protein extracts were isolated from 20 selected embryos, which were homogenized in 25 µl ECM extraction buffer (1 mM EDTA, 1,5% Triton-X 100 and 2 M urea). Samples were supplemented with 25 µl 2× SDS sample buffer, cooked at 99°C for 2 min and 20 µl were used for SDS-PAGE. Larval and adult extracts were obtained from 10 whole animals homogenized in extraction buffer. Primary antibodies were diluted in 10% dry milk powder (w/v) in TBS-T and incubated overnight at 4°C. Antibodies used were guinea pig anti-Loh (1∶5.000, this study), mouse anti-Prc/EC11 (1∶200, DSHB) and mouse anti-βTub/E7 (1∶5.000, DSHB). Alkaline phosphatase coupled secondary antibodies (Dianova, Germany) were diluted 1∶10.000 and phosphatase activity was visualized by colorimetric NBT/BCIP reaction. Total protein was stained using 0.1 µg/ml amido black 10B (Sigma) in 7% acetic acid.

### Time lapse imaging of larval heart beat

Animals were equilibrated for 20 min and heart beat was recorded on a Zeiss Axioplan upright microscope equipped with a 10× air objective (n.a. = 0.30). Single pictures were recorded at 80 frames per second (fps) using a Hamamatsu EM-CCD C9100 camera. Images were processed using Fiji and transformed into movie files.

### Dye angiography

For dye injections staged pharate adults (<90 h APF) were glued on a glass object slide using double sided scotch tape. After 10 min the operculum was removed with fine forceps to allow imaging of dye accumulation. One single injection per animal was carried out, using a glass capillary applied to a micro manipulator and an Eppendorf FemtoJet microinjector. The capillary was filled with 10 µl uranin solution (1 µg/µl in PBS) that was injected laterally into the abdomen of the animal. Dye accumulation was recorded over three minutes using a stereo microscope equipped with an UV lamp, a corresponding filter set and a consumer digital camera (Canon PowerShot A650 IS). Pixel intensities were measured using the “Plot Z-axis profile” tool of Fiji within a region of interest (R.O.I) of the head (excluding the eyes due to different pigmentation).

### Life span assay

Freshly hatched animals were collected and separated according their sex and genotype. The flies were kept in plastic vials filled with standard cornmeal agar in groups of less than 20 animals at 22°C. The number of living animals was evaluated every three to five days and the flies were transferred onto new vials.

### TEM analysis

Late stage embryos were selected according their genotype, judged by balancer expression. Fixation of embryos, sectioning and image acquisition was described previously [47]. The thickness of the basement membrane (BM) was investigated in sections of three independent animals (two sections per animal) of each genotype using Fiji. Therefore, BM thickness was measured at ten randomly picked positions in each image leading to a total number of 60 values per genotype.

### Northern blot

Northern blot was done as described previously with 15 µg total RNA loaded per lane [50]. Hybridization was carried out at 66°C for 24 h.

### Generation of UAS Loh constructs

The cDNA of *lohA* was amplified from cDNA clone GM15606 (BDGP). Oligonucleotides used were *lohA*-*Eco*RI-F TACTCAGAATTCATGGCGAAGCTGTTGTTAATATTCAG and *lohA*-*Kpn*I-R TACTCAGGTACCTTAAATGCCACCCGTGCAGGAAAAAC. The *lohA^ΔSP^* coding DNA was amplified using the modified oligonucleotide *lohA^ΔSP^*-*Eco*RI-F TACTCAGAATTCATG GATTTAACAACTAAAGAGCG. The resulting DNA fragments were cloned into the pUAST vector and transgenic flies were established after standard protocols (TheBestGene Inc., USA).

### Generation of the Loh antibody

An antiserum against Loh was generated by injecting two guinea pigs with the sequence specific peptide VFDYHRIDGAEDSNGVTEW-C bound to KLH. Harvested antiserum was affinity purified against the peptide. Peptide synthesis, serum production and affinity purification were carried out by a commercial service (Pineda Antikörperservice, Berlin).

### Immunoprecipitation

All steps were carried out at 4°C or on ice. Total protein from 100 mg adult *prc*-Gal4/+; UAS-LohA/+ flies (∼100 flies) was extracted in 500 µl ECM extraction buffer (1 mM EDTA, 1,5% Triton-X 100 and 2 M urea). Flies were homogenized, pulled 6-times through a syringe (Ø = 0,8 mm) and debris was spun down at 8.000 g for 30 min. The supernatant was centrifuged again at 13.100 g for 30 min. The soluble protein fraction was split into four 100 µl aliquots. One aliquot served as input. The other aliquots were supplemented with 10 µl Protein A-Sepahrose 4B (Sigma), 0,1% BSA and either 10 µl PBS (negative control), 10 µl anti-Loh or 67 µl anti-Prc antibody and incubated under constant shaking overnight. Protein A slurry was spun down at 13.100 g for 10 min and the pellet was washed in 500 µl ice cold 1M NaCl. The washing step was repeated three times, afterwards the pellets were resolved in 60 µl 2× SDS sample buffer and used for Western blotting

## Supporting Information

Figure S1 *loh* maps to the second chromosome and is identical to CG6232. (**A–B**) Cardiac phenotype initially identified in the EMS allele *loh^1^*. Homozygous mutant first instar larvae display a detachment of pericardial cells (arrowhead) from the heart tube (visualized by *hand*C-GFP). (**C–F**) Transheterozygous third instar larvae used for mapping of *loh^1^*. The allele fails to complement Df(2L)Exel7048, Df(2L)BSC144 and Df(2L)BSC453. (**G, H**) The gene specific allele *loh^MI02765^* displays the pericardial cell detachment phenotype in homozygous and transheterozygous condition. (**I**) Genomic region where *loh^1^* localizes. Red bars indicate deficiencies not complemented by the allele, green bar shows deficiency that is complemented by *loh^1^*. All non-complementing deficiencies overlap in a 14 kb genomic region containing three open reading frames - CG6232, *ppk10* and CG5322. (**J**) Schematic drawing showing functional domains of both Loh isoforms in comparison to murine ADAMTSL6 (TSR1 - Thrombospondin type 1 repeat). Domains and the homologous proteins were identified using the HMMER web server [51]. The percentages indicate the degree of amino acid identity in the regions restricted by the dashed lines. The headlong Y indicates the approximate binding site of the anti-Loh antibody.
(TIF)

Figure S2 Polarity of cardiac cells. (**A–D**) Localization of the cell adhesion protein FasIII (A,B) and the cortex protein αSpectrin in stage 17 embryonic hearts (C, D). Both proteins predominantly localize to the apical and lateral membranes of cardiomyocytes. This distribution is not changed in homozygous *loh^MB05750^* (**E–H**) and *prc^MB03017^* mutants (**I–L**).
(TIF)

Figure S3 Life span of mutants. Life span curves of control (*white^1118^*) or homozygous *loh^MB05750^* and *prc^MB03017^* mutants indicate a reduction of life time upon mutation of either *loh* or *prc*.
(TIF)

Figure S4 Heart autonomous function of *loh*. (**A**) RT-PCR showing *lohA* transcript levels in control embryos compared to homozygous *loh^MB05750^* embryos. Transcripts are massively reduced in the mutants, leading to decreased protein levels (compare to Figure 4D). (**B–D**) Induced knock down of *loh* activity by expression of the IR line VDRC31020 (C) targeting both transcriptional isoforms and the isoform A specific IR line NIG6232-2 (D). The expression of both lines results in a pericardial cell detachment in third instar larvae (arrowheads).
(TIF)

Figure S5 Specificity of the anti-Loh antibody. (**A–C**) Stage 17 embryos stained against Loh. A cardiac signal is present in control and heterozygous Df(2L)Exel7048/CyO,Kr>GFP embryos (arrowheads), but absent from homozygous deficient animals (C).
(TIF)

Figure S6 ECM formation in *loh* and *prc* mutants and generated revertants. (**A, B**) Prc mis-localizes in homozygous *loh^1^* and *loh^MI02765^* stage 17 embryos very similar to the phenotype observed in *loh^MB05750^* (compare to Figure 6B). (**C**) PCR using oligonucleotides flanking the inserted minos elements in *loh^MB05750^* or *prc^MB03017^*. The chosen reaction conditions only allow amplicon formation in the absence of the minos element. Subsequent sequencing of the PCR fragments proved specific excision. (**D**) The localization of Prc is restored to the wild type situation in revertant animals where the MB05750 minos element was precisely excised (double arrowhead). (**E**) Expression of Prc is restored to wild type levels in MB03017 revertant animals. (**F–H**) Compared to the control localization of Nidogen (Ndg) is normal in homozygous *loh^MB05750^* and *prc^MB03017^* mutant stage 17 embryos. (**I–K**) Compared to the control localization of secreted Laminin (Lan) trimers is normal in homozygous *loh^MB05750^* and *prc^MB03017^* mutant stage 17 embryos. (**L–N**) TEM cross sections of control and mutant embryonic hearts (stage 17). While homozygous *loh^MB05750^* mutant hearts possess a lumen similar to the control, homozygous *prc^MB03017^* animals frequently fail to seal the heart lumen at the ventral side (double headed arrow in N points to the opposing cardiomyocytes). Scale bar is 500 nm. (**O–Q**) Localization of the Slit ligand is not altered in the mutants at embryonic stage 17, excluding an involvement of the Slit/Robo signaling cascade in the lumen defect observed in homozygous *prc^MB03017^* animals.
(TIF)

Figure S7 Fat body derived Prc contributes to heart integrity. (**A–F**) Induced knock down of *prc* activity by expression of the IR line VDRC100357 (C, D) and the specific IR line VDRC41320 (E, F), either under the control of *hand*C-Gal4 (C, E) or *prc*-Gal4 (D, F). The expression of both hairpins under the control of either driver results in a pericardial cell detachment in third instar larvae. Notably, the penetrance of the phenotype is remarkably higher when the knock down is induced using *prc*-Gal4 (compare to Figure 7).
(TIF)

Figure S8 Overexpression and localization of LohA. (**A**) Total protein extracts from third instar larvae. Immunoblots probed against Loh or Prc. The ectopic overexpression of LohA does not induce alterations in Prc protein levels. (**B**) Optical section through the fat body of a *prc*>LohA third instar larva stained against Loh (red), βPS integrin (green) and GFP (blue). Loh co-localizes to integrin in a spotty fashion along the cell contacts of adipocytes.
(TIF)

Movie S1 Heart beat pattern in a wild type semi-dissected third instar larva. Anterior is to the left.
(MP4)

Movie S2 Heart beat pattern in a transheterozygous *loh^MB05750^*/Df(2L)Exel7048 semi-dissected third instar larva. Anterior is to the left.
(MP4)

Movie S3 Heart beat pattern in a transheterozygous *prc^MB03017^*/Df(3L)*vin6* semi-dissected third instar larva. Anterior is to the left.
(MP4)

Movie S4 Accumulation of the tracer dye uranin in the head of an injected wild type pharate adult. Anterior is to the top.
(MP4)
