# Supplementary figures and images for: The Conserved ADAMTS-like Protein Lonely heart Mediates Matrix Formation and Cardiac Tissue Integrity
Source: PLoS Genet. 2013 Jul 11;9(7):e1003616. doi: 10.1371/journal.pgen.1003616 (PMC3708815; doi:10.1371/journal.pgen.1003616)

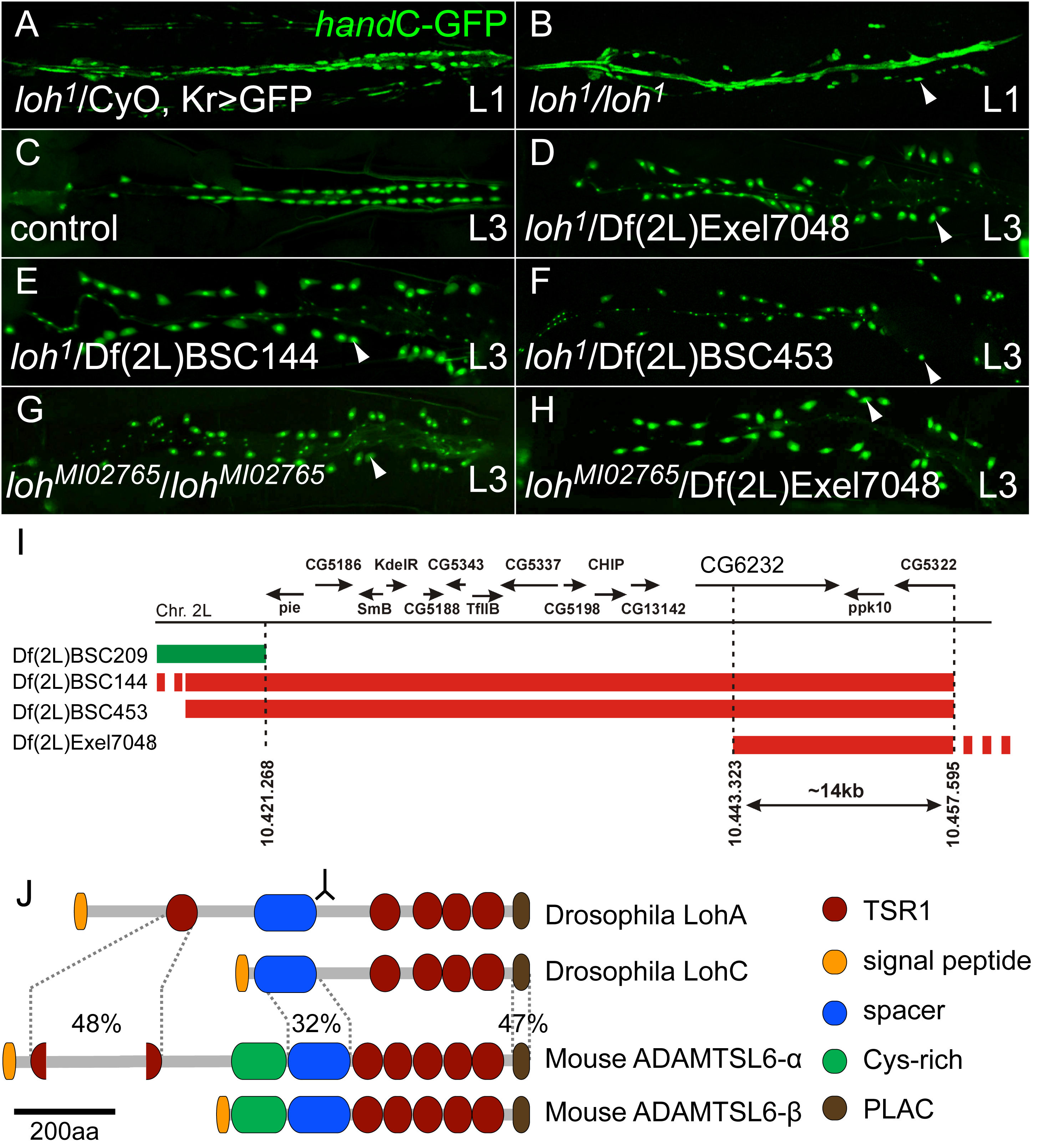

Supplement: Figure S1 — loh maps to the second chromosome and is identical to CG6232. (A–B) Cardiac phenotype initially identified in the EMS allele loh1. Homozygous mutant first instar larvae display a detachment of pericardial cells (arrowhead) from the heart tube (visualized by handC-GFP). (C–F) Transheterozygous third instar larvae used for mapping of loh1. The allele fails to complement Df(2L)Exel7048, Df(2L)BSC144 and Df(2L)BSC453. (G, H) The gene specific allele lohMI02765 displays the pericardial cell detachment phenotype in homozygous and transheterozygous condition. (I) Genomic region where loh1 localizes. Red bars indicate deficiencies not complemented by the allele, green bar shows deficiency that is complemented by loh1. All non-complementing deficiencies overlap in a 14 kb genomic region containing three open reading frames - CG6232, ppk10 and CG5322. (J) Schematic drawing showing functional domains of both Loh isoforms in comparison to murine ADAMTSL6 (TSR1 - Thrombospondin type 1 repeat). Domains and the homologous proteins were identified using the HMMER web server [51]. The percentages indicate the degree of amino acid identity in the regions restricted by the dashed lines. The headlong Y indicates the approximate binding site of the anti-Loh antibody. (TIF) [file pgen.1003616.s001.tif]

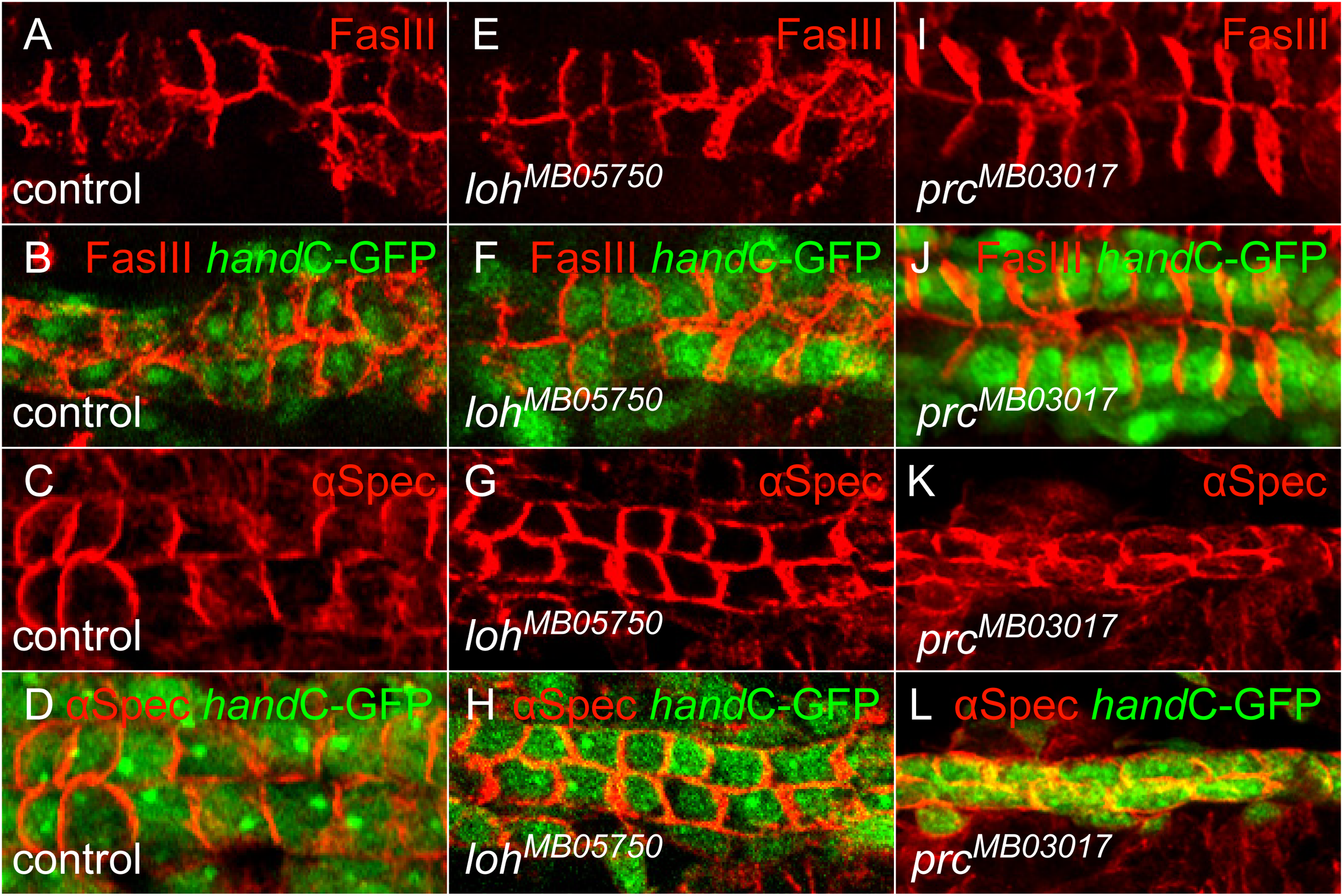

Supplement: Figure S2 — Polarity of cardiac cells. (A–D) Localization of the cell adhesion protein FasIII (A,B) and the cortex protein αSpectrin in stage 17 embryonic hearts (C, D). Both proteins predominantly localize to the apical and lateral membranes of cardiomyocytes. This distribution is not changed in homozygous lohMB05750 (E–H) and prcMB03017 mutants (I–L). (TIF) [file pgen.1003616.s002.tif]

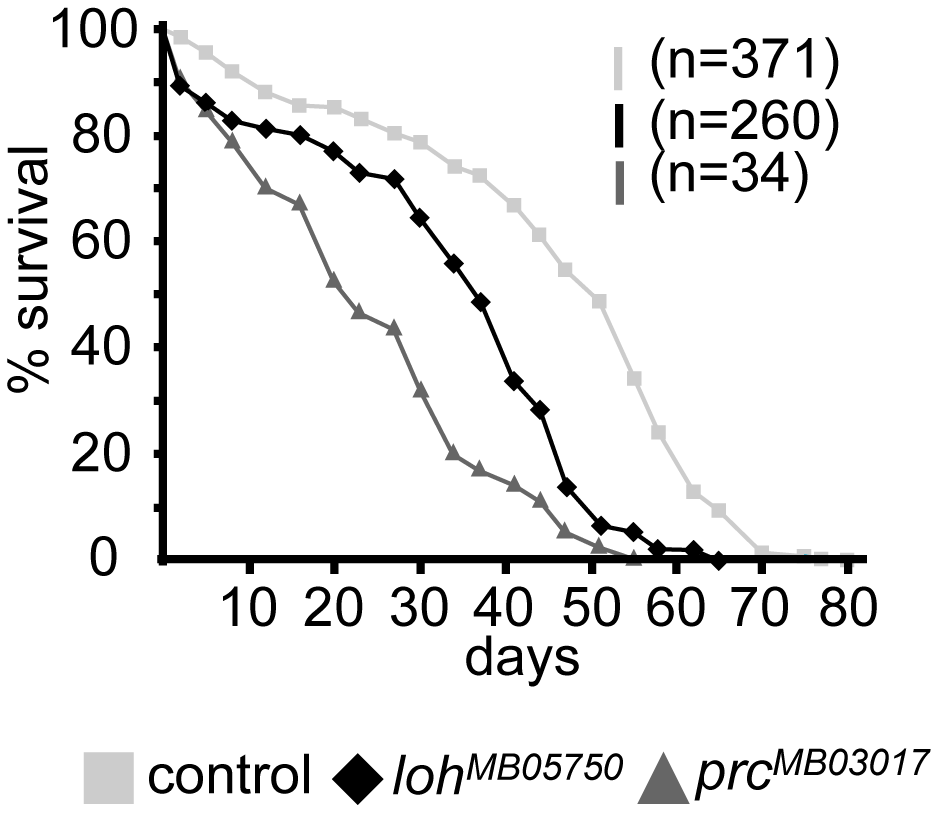

Supplement: Figure S3 — Life span of mutants. Life span curves of control (white1118) or homozygous lohMB05750 and prcMB03017 mutants indicate a reduction of life time upon mutation of either loh or prc. (TIF) [file pgen.1003616.s003.tif]

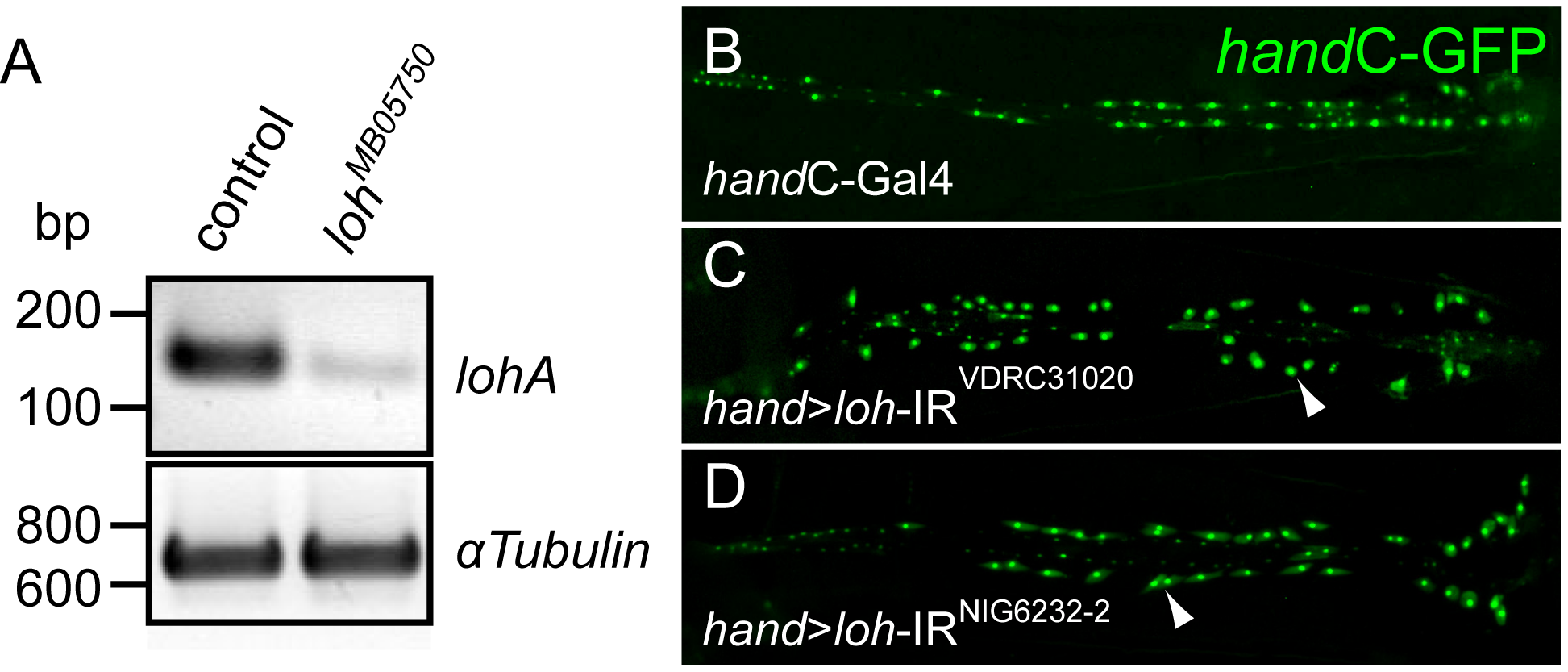

Supplement: Figure S4 — Heart autonomous function of loh. (A) RT-PCR showing lohA transcript levels in control embryos compared to homozygous lohMB05750 embryos. Transcripts are massively reduced in the mutants, leading to decreased protein levels (compare to Figure 4D). (B–D) Induced knock down of loh activity by expression of the IR line VDRC31020 (C) targeting both transcriptional isoforms and the isoform A specific IR line NIG6232-2 (D). The expression of both lines results in a pericardial cell detachment in third instar larvae (arrowheads). (TIF) [file pgen.1003616.s004.tif]

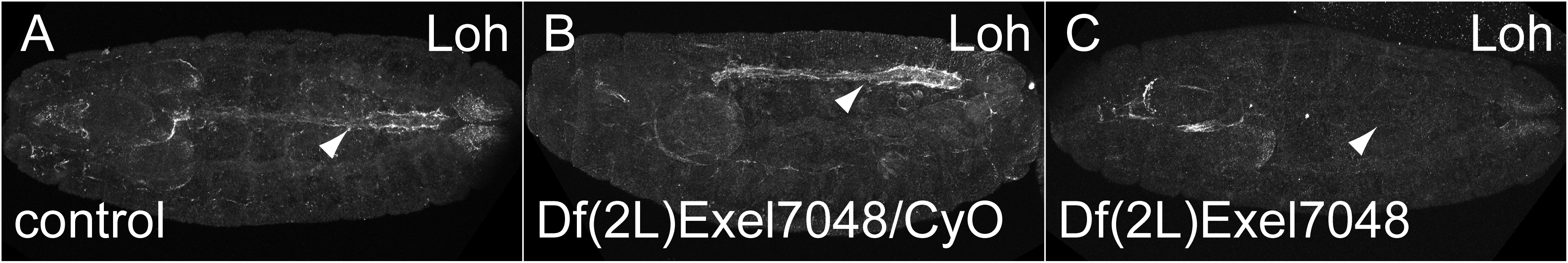

Supplement: Figure S5 — Specificity of the anti-Loh antibody. (A–C) Stage 17 embryos stained against Loh. A cardiac signal is present in control and heterozygous Df(2L)Exel7048/CyO,Kr>GFP embryos (arrowheads), but absent from homozygous deficient animals (C). (TIF) [file pgen.1003616.s005.tif]

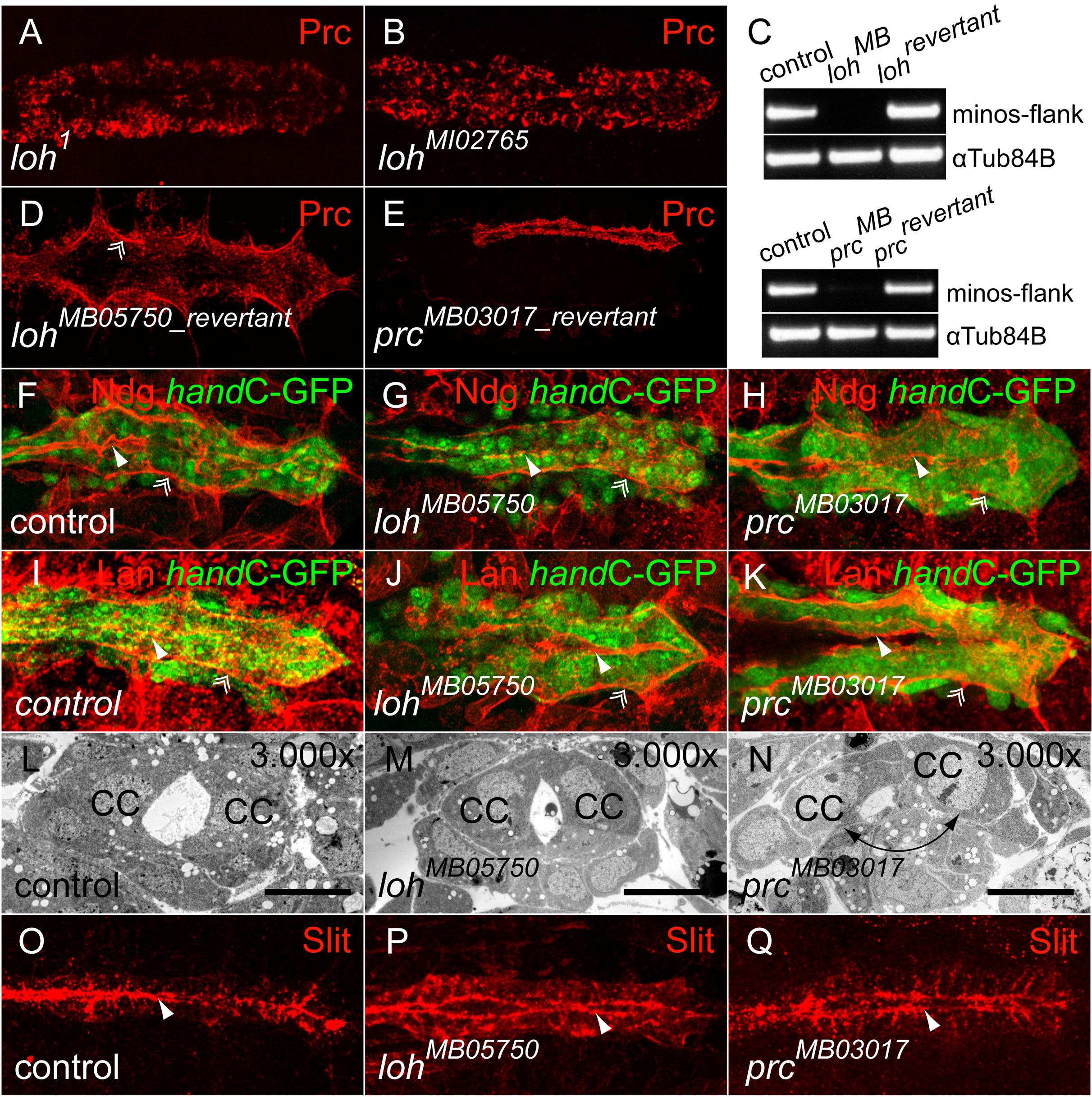

Supplement: Figure S6 — ECM formation in loh and prc mutants and generated revertants. (A, B) Prc mis-localizes in homozygous loh1 and lohMI02765 stage 17 embryos very similar to the phenotype observed in lohMB05750 (compare to Figure 6B). (C) PCR using oligonucleotides flanking the inserted minos elements in lohMB05750 or prcMB03017. The chosen reaction conditions only allow amplicon formation in the absence of the minos element. Subsequent sequencing of the PCR fragments proved specific excision. (D) The localization of Prc is restored to the wild type situation in revertant animals where the MB05750 minos element was precisely excised (double arrowhead). (E) Expression of Prc is restored to wild type levels in MB03017 revertant animals. (F–H) Compared to the control localization of Nidogen (Ndg) is normal in homozygous lohMB05750 and prcMB03017 mutant stage 17 embryos. (I–K) Compared to the control localization of secreted Laminin (Lan) trimers is normal in homozygous lohMB05750 and prcMB03017 mutant stage 17 embryos. (L–N) TEM cross sections of control and mutant embryonic hearts (stage 17). While homozygous lohMB05750 mutant hearts possess a lumen similar to the control, homozygous prcMB03017 animals frequently fail to seal the heart lumen at the ventral side (double headed arrow in N points to the opposing cardiomyocytes). Scale bar is 500 nm. (O–Q) Localization of the Slit ligand is not altered in the mutants at embryonic stage 17, excluding an involvement of the Slit/Robo signaling cascade in the lumen defect observed in homozygous prcMB03017 animals. (TIF) [file pgen.1003616.s006.tif]

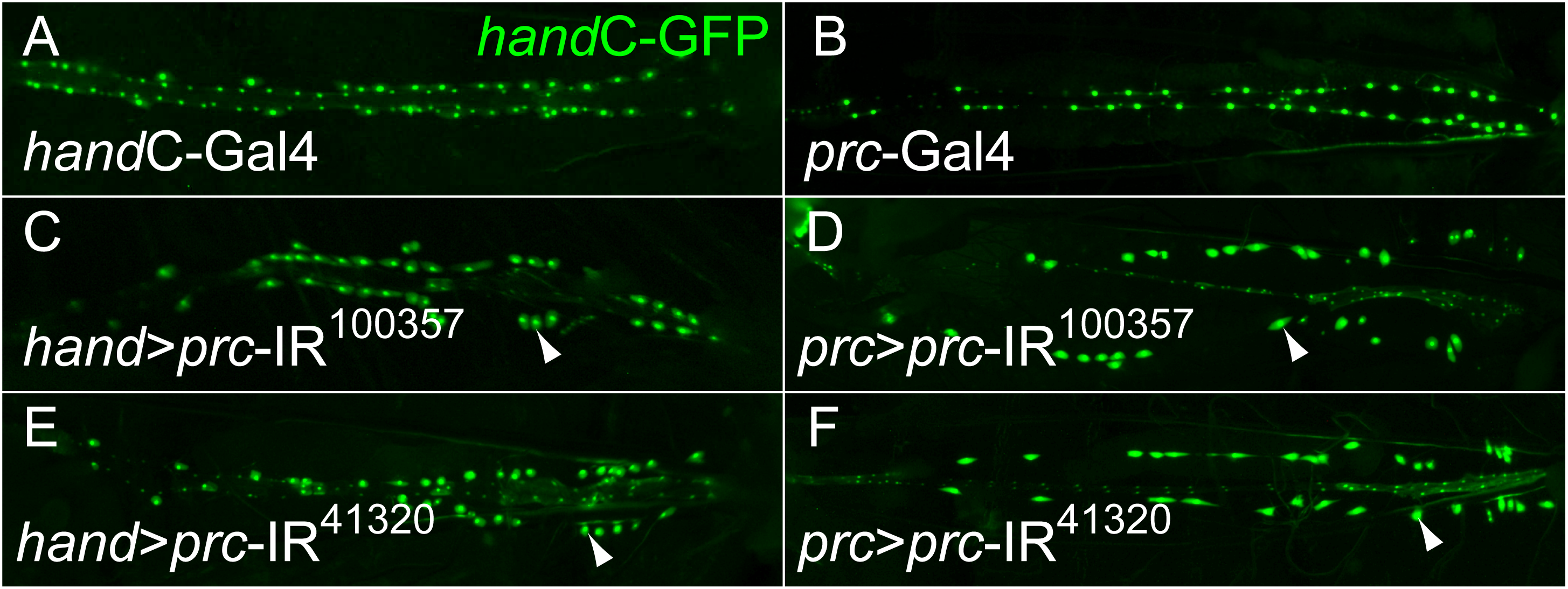

Supplement: Figure S7 — Fat body derived Prc contributes to heart integrity. (A–F) Induced knock down of prc activity by expression of the IR line VDRC100357 (C, D) and the specific IR line VDRC41320 (E, F), either under the control of handC-Gal4 (C, E) or prc-Gal4 (D, F). The expression of both hairpins under the control of either driver results in a pericardial cell detachment in third instar larvae. Notably, the penetrance of the phenotype is remarkably higher when the knock down is induced using prc-Gal4 (compare to Figure 7). (TIF) [file pgen.1003616.s007.tif]

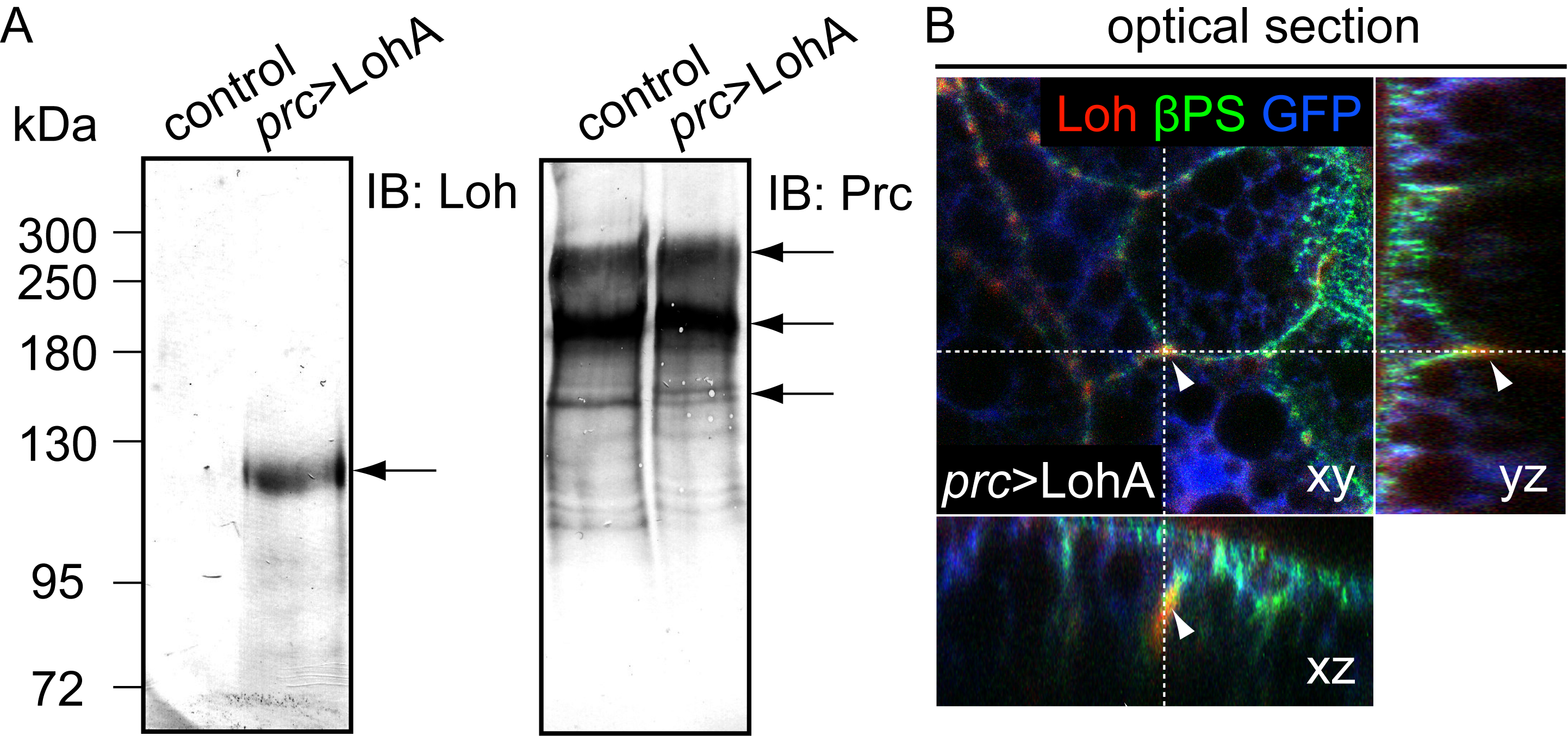

Supplement: Figure S8 — Overexpression and localization of LohA. (A) Total protein extracts from third instar larvae. Immunoblots probed against Loh or Prc. The ectopic overexpression of LohA does not induce alterations in Prc protein levels. (B) Optical section through the fat body of a prc>LohA third instar larva stained against Loh (red), βPS integrin (green) and GFP (blue). Loh co-localizes to integrin in a spotty fashion along the cell contacts of adipocytes. (TIF) [file pgen.1003616.s008.tif]
